# CARD9 Conveys Pancreatic Islet Sympathetic Nervous β2 Signals to Reshape Macrophage Creatine Metabolism in Type 1 Diabetes

**DOI:** 10.1002/advs.202507543

**Published:** 2025-11-16

**Authors:** Huimin Yuan, Senlin Li, Shuai Liu, Hong Zhou, Yue Xu, Wenhui Li, Mengxuan Wang, Mengheng Wang, Mengzhu Zheng, Wenxi Shi, Xin Li, Yiting Feng, Ming Xiang

**Affiliations:** ^1^ Department of Pharmacology School of Pharmacy Tongji Medical College Huazhong University of Science and Technology Wuhan 430000 China; ^2^ Tongji Hospital Tongji Medical College Huazhong University of Science and Technology Wuhan 430000 China; ^3^ Department of Pharmacy Union Hospital Tongji Medical College Huazhong University of Science and Technology Wuhan 430022 China; ^4^ Institute of Pharmaceutical Innovation Hubei Province Key Laboratory of Occupational Hazard Identification and Control School of Medicine Wuhan University of Science and Technology Wuhan 430065 China

**Keywords:** β2‐AR, CARD9, creatine, macrophage, sympathetic nerve, type 1 diabetes

## Abstract

Type 1 diabetes (T1D) is an autoimmune disorder marked by the injury of pancreatic β cells, during which sympathetic neurons in the endocrine region of pancreas are lost, whereas those in the exocrine regions surrounding islets remain intact. This abnormal sympathetic nervous signaling may disrupt the balance of the neuroendocrine‐immune network and contribute to the development of T1D, although its underlying molecular mechanisms are still elusive. Here, single‐cell omics and whole‐tissue immunostaining reveal a decrease in pancreatic sympathetic nerve density in T1D patients and T1D mouse models. Surgical and chemical desensitization of the sympathetic nervous system exacerbates T1D, while macrophage depletion mitigates this effect. Mechanistically, diminished norepinephrine (NE) release impairs β2‐adrenergic receptor (β2‐AR)‐PKA‐CREB1 signaling in islet macrophages, leading to downregulation of the adaptor caspase recruitment domain family member 9 (CARD9). Loss of CARD9 decreases SLC6A8‐mediated creatine uptake, shifts macrophages toward a pro‐inflammatory phenotype, and promotes sympathetic axon ferroptosis driven by decreased neurotrophic factor and anti‐inflammatory factor release. Conversely, β2‐AR agonist formoterol restores PKA‐CREB1‐CARD9 activation, preserves creatine metabolism, and maintains anti‐inflammatory macrophage polarization. These findings define a novel sympathetic‐macrophage‐creatine metabolic axis governed by CARD9 that links neural signals to immune and metabolic regulation in T1D, highlighting neuroimmune interactions as targets for therapies.

## Graphical abstract

This study identifies CARD9 as a key mediator linking sympathetic β2‐adrenergic receptor signaling to macrophage creatine metabolism, inflammatory polarization, and neuronal integrity. Loss of β2‐AR‐PKA‐CREB1‐CARD9 signaling in macrophages reduces creatine uptake, promotes pro‐inflammatory macrophage activation, and drives sympathetic axon ferroptosis. Disruption of this neuro‐immune axis accelerates β‐cell autoimmunity and T1D progression, establishing CARD9 as a central regulator of macrophage metabolism, inflammation, and axon survival.

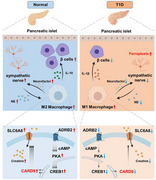

## 1 Introduction

Type 1 diabetes (T1D) is a chronic autoimmune disease characterized by targeted destruction of pancreatic β cells by the immune system.^[^ ^1^ ^]^ Within the pancreas, the sympathetic nervous system plays a multifaceted regulatory role, including direct modulation of endocrine cell function, control of local blood flow via vascular vasoconstrictor cells, and interaction with pancreatic immune cells to maintain homeostasis.^[^ ^2^ ^]^ Interestingly, during early‐stage of T1D, sympathetic neurons specifically within the endocrine regions of the pancreas undergo selective loss, while those in exocrine regions remain intact.^[^ ^3^, ^4^ ^]^ This regional denervation may destabilize the neuro‐immune‐endocrine network, exacerbating both autoimmune attack on β cells and local neurodegeneration.

Macrophages in the pancreas have close contact with sympathetic neurons and may be involved in the transmission of neuroimmune signals during the development of T1D.^[^ ^5^, ^6^ ^]^ Macrophages play a pivotal role in the inflammatory infiltration of islets and neurons in T1D. As antigen‐presenting cells, they process β‐cell antigens and present them to T cells, triggering autoimmune responses, while also releasing pro‐inflammatory factors interleukin‐1 beta (IL‐1β) and tumor necrosis factor‐alpha (TNF‐α), which directly damage β cells.^[^ ^1^, ^7^ ^]^ Moreover, macrophage‐derived factors such as inflammatory factors and neurotrophic factors can promote lipid peroxidation in neighboring neurons, sensitizing them to ferroptosis under conditions of iron overload. Conversely, secretion of anti‐oxidative mediators by alternatively‐activated macrophages can inhibit neuronal ferroptosis, thus modulating neuroimmune balance.

Caspase recruitment domain family member 9 (CARD9), which is highly expressed in immune cells such as macrophages and dendritic cells (DCs), functions in both innate and adaptive immunity.^[^ ^8^ ^]^ As a central signaling hub, CARD9 enables the integration of various signals, activation of downstream molecular cascades, and regulation of cellular metabolism to maintain homeostasis.^[^ ^9^, ^10^, ^11^ ^]^ It has been implicated in multiple diseases, including neurodegenerative diseases, infections, cancer, inflammatory disorders, and metabolic conditions.^[^ ^12^, ^13^ ^]^ However, there is an urgent need for the exploration of CARD9‐specific role in T1D and neuropathy.

We previously found that the expression level of CARD9 is closely related to the intracellular creatine (Cr) metabolism and its transporter, solute carrier family 6 member 8 (SLC6A8) expression. Cr, a nitrogen‐containing amino acid, plays a crucial role in modulating macrophage polarization and exerting neuroprotective effects.^[^ ^14^ ^]^ Cr supplementation has been shown to suppress the janus kinase (JAK)‐ signal transducer and activator of transcription 1 (STAT1)‐ inducible nitric oxide synthase (iNOS) signaling pathway, thereby attenuating pro‐inflammatory macrophage activation. Concurrently, it enhances the IL‐4‐STAT6‐arginase 1 (ARG1) axis, promoting anti‐inflammatory macrophage polarization.^[^ ^15^ ^]^ Beyond its immunomodulatory functions, Cr contributes to neuronal energy metabolism by rapidly regenerating ATP via the phosphocreatine (PCr) system, ensuring sufficient energy supply for synaptic activity.^[^ ^16^, ^17^ ^]^

In this study, we discovered that the pancreatic sympathetic nervous system undergoes selective degeneration, concurrent with dynamic macrophage remodeling. Both chemical and surgical suppression of sympathetic nervous activity worsened T1D progression, whereas macrophage depletion counteracted this effect. CARD9 acted as a key downstream effector of sympathetic β2‐AR‐PKA‐CREB1 signaling in macrophages. When CARD9 was downregulated, SLC6A8‐mediated Cr uptake was impaired, driving macrophages toward a pro‐inflammatory phenotype and increasing the secretion of pro‐inflammatory cytokines that accelerated T1D progression. Furthermore, macrophage CARD9 influenced sympathetic nerve loss and promoted ferroptosis by modulating inflammatory cytokines and neurotrophic factor production. In contrast, pharmacological activation of β2‐AR with formoterol reinstated PKA‐CREB1‐CARD9 signaling, enhanced Cr uptake, and promoted anti‐inflammatory macrophage activation. These findings elucidated a novel mechanism of neural‐immune network dysregulation in T1D and neuropathy pathogenesis, underscoring potential neuroprotective approaches for developing new therapeutic interventions.

## 2 Results

### 2.1 Specific Loss of Islet Sympathetic Nerves during T1D

**Figure 1:**
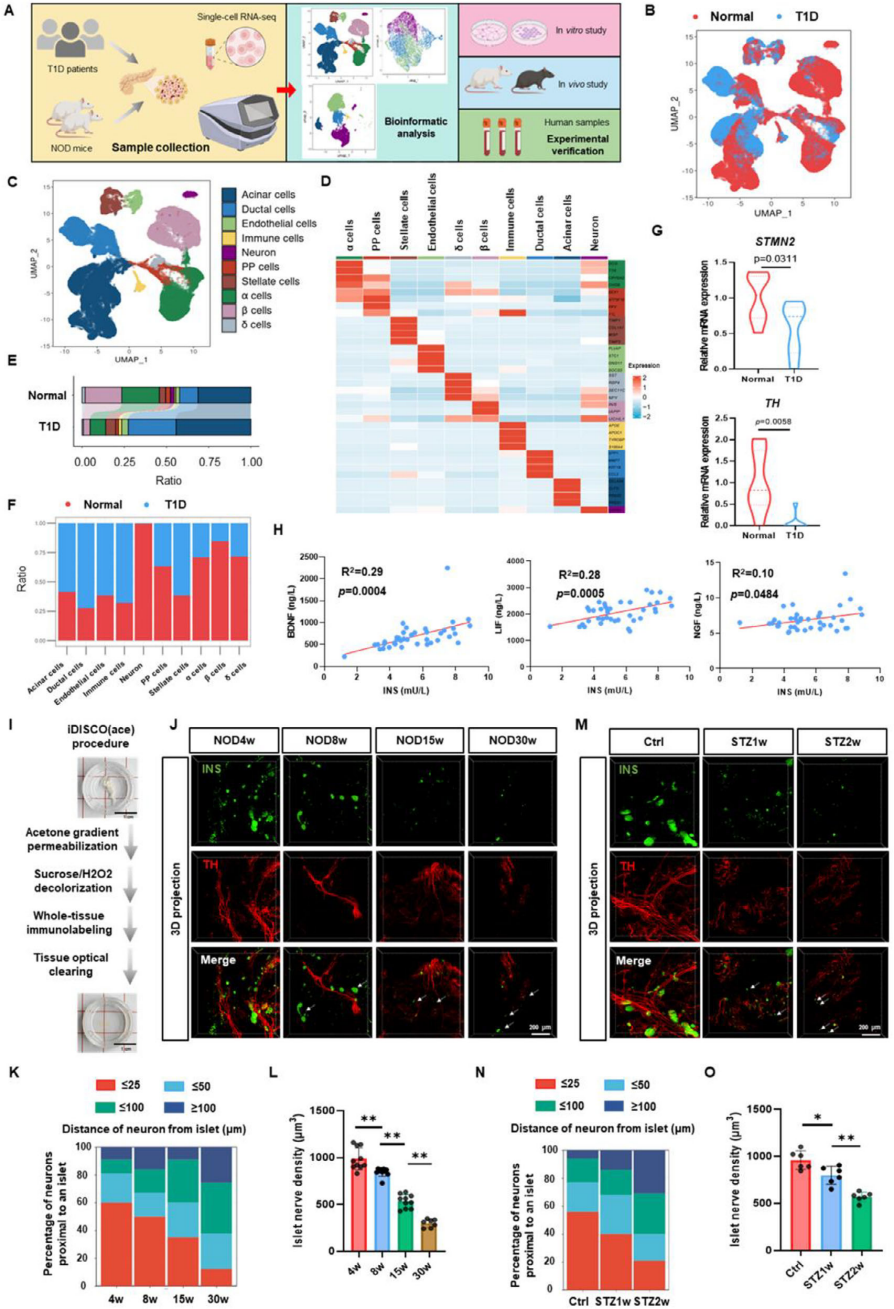
Selective loss of islet sympathetic nerves during T1D. A) Schematic overview of the study design. Single‐cell transcriptome data generated on the 10× Genomics platform were analyzed using R. The scRNA‐seq data of pancreatic tissue from T1D patients were obtained from GSE148073, and immune cell data from NOD mouse pancreatic tissue were retrieved from GSE141784 (GSM4213196 to GSM4213200). The transcriptomic data were comprehensively analyzed and validated through in vivo and in vitro experiments, as well as clinical sample analysis. B) UMAP visualization of pancreatic single cells from healthy individuals and T1D patients. C) UMAP visualization of 10 distinct cell clusters in the pancreas of T1D patients. D) Heatmap showing the average expression levels of known markers across 10 cell types. Color intensity indicates the proportion of cells expressing specific genes within each cell type. E, F) Bar charts illustrating the proportions of 10 cell clusters in healthy individuals and T1D patients. G) Bar chart depicting the expression levels of *STMN2* and *TH* in pancreatic tissues from healthy individuals and T1D patients. H) Scatter plot illustrating the correlations between insulin and BDNF, LIF, and NGF in the plasma of diabetes patients. I) Workflow of pancreatic tissue clearing for transparent imaging. J, K) Whole‐tissue immunofluorescence of pancreatic tissues from female NOD mice at different ages. Insulin is shown in green, and TH in red. The distances between sympathetic nerve fibers and pancreatic islets were analyzed and quantified using Imaris software. L) Quantification of islet sympathetic nerve fiber density in female NOD mice at different ages using Imaris software (n = 10). M, N) Whole‐tissue immunofluorescence of pancreatic tissues from male C57BL/6J mice at various time points after STZ injection to induce T1D. Insulin is shown in green, and TH in red. The distances between sympathetic nerve fibers and pancreatic islets were analyzed and quantified using Imaris software. O) Quantification of islet sympathetic nerve fiber density in male C57BL/6J mice at different time points after STZ injection using Imaris software (*n* = 6). The data were compiled from at least three independent experiments and are presented as the means ± SEMs. ^*^ *p* < 0.05, ^**^ *p* < 0.01, ^***^ *p* < 0.001 by Student's unpaired two‐tailed t test or one‐way analysis of variance (ANOVA) with Tukey's post hoc comparison.

To elucidate the mechanisms underlying islet sympathetic dysregulation in T1D, we conducted a comprehensive investigation integrating single‐cell sequencing analysis, in vivo and in vitro experiments, and clinical sample validation (**Figure** **1**A). Utilizing the Seurat package, we first integrated single‐cell data from T1D patients and healthy human islets. UMAP clustering identified ten distinct cell populations: acinar, ductal, endothelial, immune, neural, pancreatic polypeptide (PP), astrocytes, alpha, beta, and delta cells (Figure 1B–D). Comparative analysis revealed significant dynamic changes in cell type proportions, particularly a marked reduction in β cells and neural cells, accompanied by an increase in ductal cells (Figure 1E,F). Transcriptomic analysis demonstrated a significant downregulation of sympathetic nervous markers *tyrosine hydroxylase (TH)* and *stathmin 2 (STMN2)* in T1D pancreatic islets. In contrast, the vagus nerve markers *choline O‐acetyltransferase (ChAT)* and *vesicular acetylcholine transporter (VAChT)*, as well as the sensory nerve marker calcitonin *gene‐related peptide alpha (CGRP)*, showed no significant changes and maintained relatively low expression levels (Figure 1G; Figure S1A, Supporting Information). Clinical correlation analysis further revealed positive associations between serum insulin (INS) levels and neurotrophic factors, including brain‐derived neurotrophic factor (BDNF), leukemia inhibitory factor (LIF), nerve growth factor (NGF), and ciliary neurotrophic factor (CNTF) (Figure 1H; Figure S1B,C, Supporting Information).

Next, we employed whole‐organ transparency techniques for 3D imaging of pancreatic tissues from female NOD mice and male STZ‐induced T1D mice (Figure 1I; Videos S1 and S2). The results indicated that pancreatic islets in both NOD and STZ‐treated mice exhibited loose and atrophic structures during diabetes progression. The number of TH^+^ neurons was markedly reduced, the distance between islets and sympathetic nerve terminals was increased, and the density of sympathetic nerve fibers within the islets was significantly decreased (Figure 1J–O). Molecular analysis corroborated these findings, showing decreased expression of neuronal markers *growth‐associated protein 43 (Gap43)*, *tubulin beta 3 (Tubb3)*, and *Stmn2*, along with reduced TH protein levels in pancreatic tissues of both mouse models (Figure S1D,E, Supporting Information). To assess whether these neural changes are sex‐dependent, we analyzed both male and female mice. In NOD mice, although diabetes incidence was lower in males than in females, diabetic male mice displayed comparable loss of islet sympathetic innervation and reduced expression of *Gap43*, *Tubb3*, and *Stmn2* (Figure S2A–D, Supporting Information). In the STZ model, similar reductions in sympathetic innervation and neuronal marker expression were observed in female mice, consistent with the male data (Figure S2E–H, Supporting Information). Together, these findings indicate an early and selective loss of islet sympathetic innervation during T1D progression, and importantly, this phenomenon is not sex‐dependent.

Importantly, our additional experiments demonstrate that islet sympathetic nerve loss is not a secondary consequence of hyperglycemia or hypoinsulinemia. In nonautoimmune hyperglycemic mouse models, including *db/db* mice and high‐fat diet–induced hyperglycemic mice, no sympathetic denervation was detected; instead, islet innervation exhibited a slight increase (Figure S3A–H, Supporting Information). In STZ‐induced T1D mice, daily insulin glargine supplementation normalized blood glucose levels but failed to restore islet sympathetic innervation (Figure S3I–L, Supporting Information).

### 2.2 Inhibition of Sympathetic Adrenergic Signaling Exacerbates T1D

**Figure 2:**
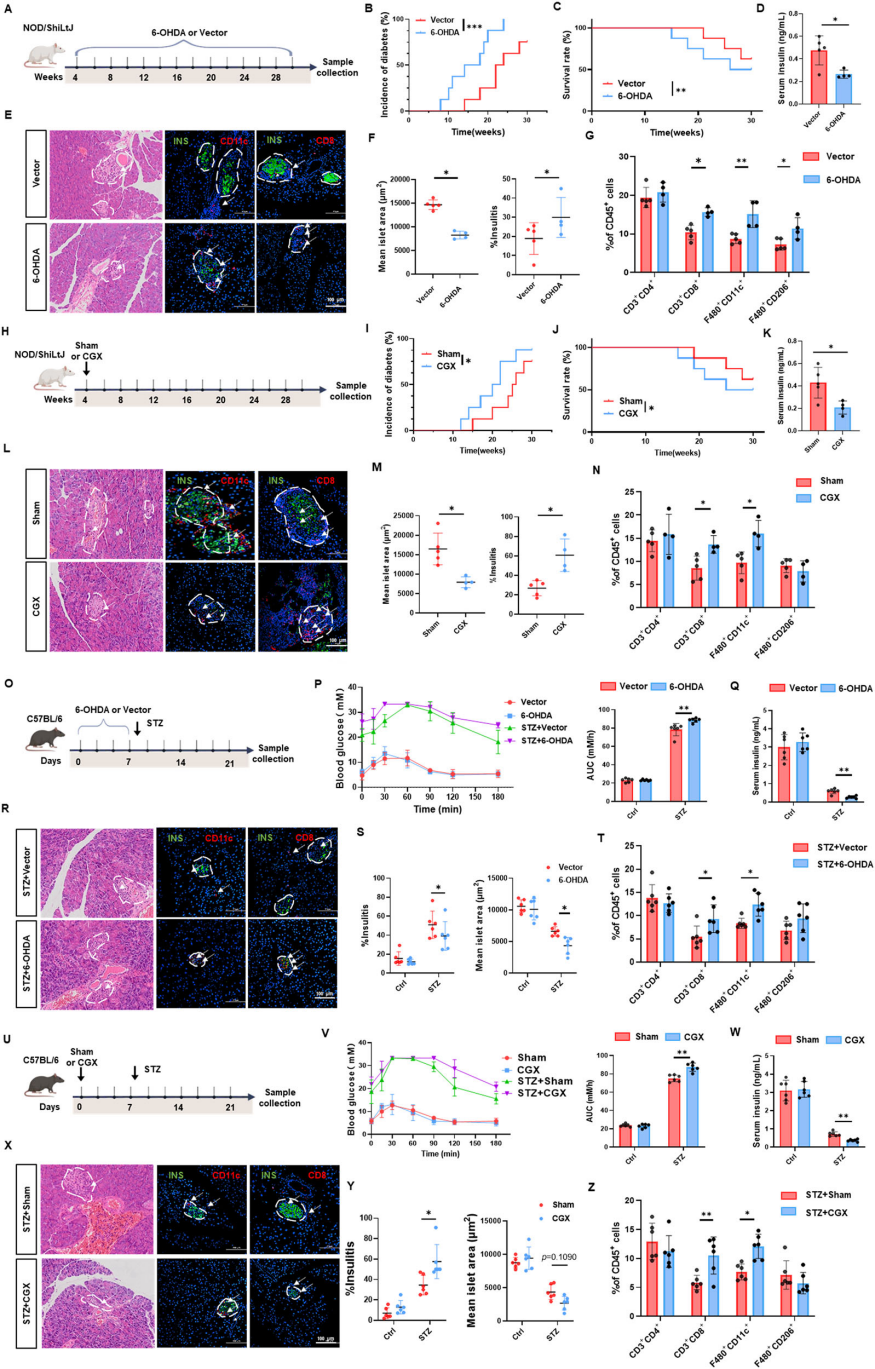
Inhibition of sympathetic adrenergic signaling exacerbates T1D. A) Flowchart of the mouse experimental design. NOD mice were divided into two groups: one group received intraperitoneal injections of 6‐hydroxydopamine (6‐OHDA), while the control group received physiological saline containing 0.1% L‐ascorbic acid injections (*n* = 8). B) Incidence of T1D. C) Survival rates. D) Plasma insulin levels. E) Representative H&E staining and immunofluorescence images illustrating insulin (INS), CD11c, and CD8 expression in pancreatic tissues. F) Quantitative analysis of average pancreatic islet area and insulitis. G) Flow cytometry analysis of immune cell populations in pancreatic tissues. The proportions of CD3^+^CD4^+^, CD3^+^CD8^+^, F4/80^+^CD11c^+^, and F4/80^+^CD206^+^ cells were quantified in each group. H) Schematic diagram of the mouse experiment. NOD mice were divided into two groups: one underwent CGX surgery, while the control group underwent sham surgery (*n* = 8). I) Incidence of T1D. J) Survival rates. K) Plasma insulin levels. L) Representative H&E staining and immunofluorescence images illustrating INS, CD11c, and CD8 expression in pancreatic tissues. M) Quantitative analysis of average pancreatic islet area and insulitis. N) Flow cytometry analysis of immune cell populations in pancreatic tissues. The proportions of CD3^+^CD4^+^, CD3^+^CD8^+^, F4/80^+^CD11c^+^, and F4/80^+^CD206^+^ cells were quantified in each group. O) Flowchart of the mouse experimental design. C57BL/6J mice were divided into four groups, receiving intraperitoneal injections of either STZ or citrate buffer, combined with physiological saline containing 0.1% L‐ascorbic acid or 6‐OHDA treatments. (*n* = 6). P) Oral glucose tolerance test (OGTT) on day 21. Q) Plasma insulin levels. R) Representative H&E staining and immunofluorescence images illustrating INS, CD11c, and CD8 expression in pancreatic tissues. S) Quantitative analysis of average pancreatic islet area and insulitis. T) Flow cytometry analysis of immune cell populations in pancreatic tissues. The proportions of CD3^+^CD4^+^, CD3^+^CD8^+^, F4/80^+^CD11c^+^, and F4/80^+^CD206^+^ cells were quantified in each group. U) Schematic diagram of the mouse experiment. C57BL/6J mice were divided into four groups: one underwent CGX surgery, while the control group underwent sham surgery. Mice were injected with either STZ or citrate buffer for observation (*n* = 6). V) OGTT on day 21. W) Plasma insulin levels. X) Representative H&E staining and immunofluorescence images illustrating INS, CD11c, and CD8 expression in pancreatic tissues. Y) Quantitative analysis of average pancreatic islet area and insulitis. Z) Flow cytometry analysis of immune cell populations in pancreatic tissues. The proportions of CD3^+^CD4^+^, CD3^+^CD8^+^, F4/80^+^CD11c^+^, and F4/80^+^CD206^+^ cells were quantified in each group. The data were compiled from at least three independent experiments and are presented as the means ± SEMs. ^*^ *p* < 0.05, ^**^ *p* < 0.01, ^***^ *p* < 0.001 by Student's unpaired two‐tailed t test or one‐way analysis of variance (ANOVA) with Tukey's post hoc comparison.

To investigate the contribution of sympathetic dysfunction to the progression of T1D, we assessed the effects of sympathetic inhibition on disease development using the neurotoxin 6‐hydroxydopamine (6‐OHDA) (**Figure** **2**A; Figure S4A, Supporting Information). 6‐OHDA selectively targets NE neurons by disrupting the amine uptake mechanism at sympathetic nerve terminals, while sparing dopaminergic (DA).^[^ ^18^, ^19^ ^]^ As a result, NE release from sympathetic nerve endings is impaired, leading to reduced sympathetic drive to downstream targets.^[^ ^20^ ^]^ In the NOD mouse model, 6‐OHDA treatment significantly increased diabetes incidence and mortality, accompanied by reduced serum insulin levels (Figure 2B–D). Histological analysis further revealed enhanced immune cell infiltration into pancreatic islets and a reduction in islet area (Figure 2E,F). Annexin V‐FITC/PI staining demonstrated increased apoptosis within islet tissues following 6‐OHDA administration (Figure S4B, Supporting Information). Flow cytometric analysis of pancreatic tissue, peripheral blood, and spleen indicated a significant rise in CD11c^+^ and CD206^+^ macrophages as well as CD8^+^ T cells in the pancreas, along with elevated CD4^+^ T cells in the spleen (Figure 2G; Figure S4C, Supporting Information).

Additionally, we inhibited sympathetic activity surgically via celiac ganglionectomy (CGX) (Figure 2H; Figure S4D, Supporting Information). CGX directly disrupts sympathetic nerve input by removing the sympathetic ganglia located at the root of the abdominal aorta, resulting in a marked reduction of sympathetic activity.^[^ ^21^ ^]^ Similar to 6‐OHDA treatment, CGX significantly elevated diabetes incidence and mortality and lowered serum insulin levels in NOD mice (Figure 2I–K). Histological evaluation showed increased immune infiltration and a reduction in islet size (Figure 2L,M), as well as elevated islet apoptosis (Figure S4E, Supporting Information). Flow cytometry confirmed that CGX also promoted the accumulation of CD11c^+^ and CD8^+^ T cells in pancreatic tissue, and increased CD8^+^ T cells in the blood (Figure 2N; Figure S4F, Supporting Information).

To further validate the impact of sympathetic inhibition on T1D progression, both 6‐OHDA treatment or CGX surgery were applied in the STZ‐induced T1D mouse model (Figure 2O,U; Figure S5A,F, Supporting Information). Consistent with the NOD model, 6‐OHDA accelerated diabetes onset, caused significant weight loss, and led to hyperglycemia (Figure S5B,C, Supporting Information). Oral glucose tolerance tests (OGTT) confirmed impaired glucose clearance, and ELISA revealed reduced serum insulin levels (Figure 2P,Q). Histological analysis demonstrated increased immune cell infiltration and diminished islet area (Figure 2R,S), as well as enhanced islet cell apoptosis (Figure S5D, Supporting Information). Flow cytometry showed increased infiltration of CD11c^+^ macrophages and CD8^+^ T cells in the pancreas, along with elevated CD4^+^ and CD8^+^ T cell populations in the spleen (Figure 2T; Figure S5E, Supporting Information). Similarly, CGX aggravated the severity of STZ‐induced T1D, further supporting the notion that suppression of sympathetic activity accelerates disease progression (Figure 2U–Z; Figure S5F–J, Supporting Information).

Overall, these findings indicate that early disruption of sympathetic adrenergic signaling promotes immune cell infiltration and β‐cell destruction, thereby accelerating the onset and progression of T1D.

### 2.3 Macrophages within Pancreatic Islets are Closely Correlated with Sympathetic Nerve Signals in T1D

**Figure 3:**
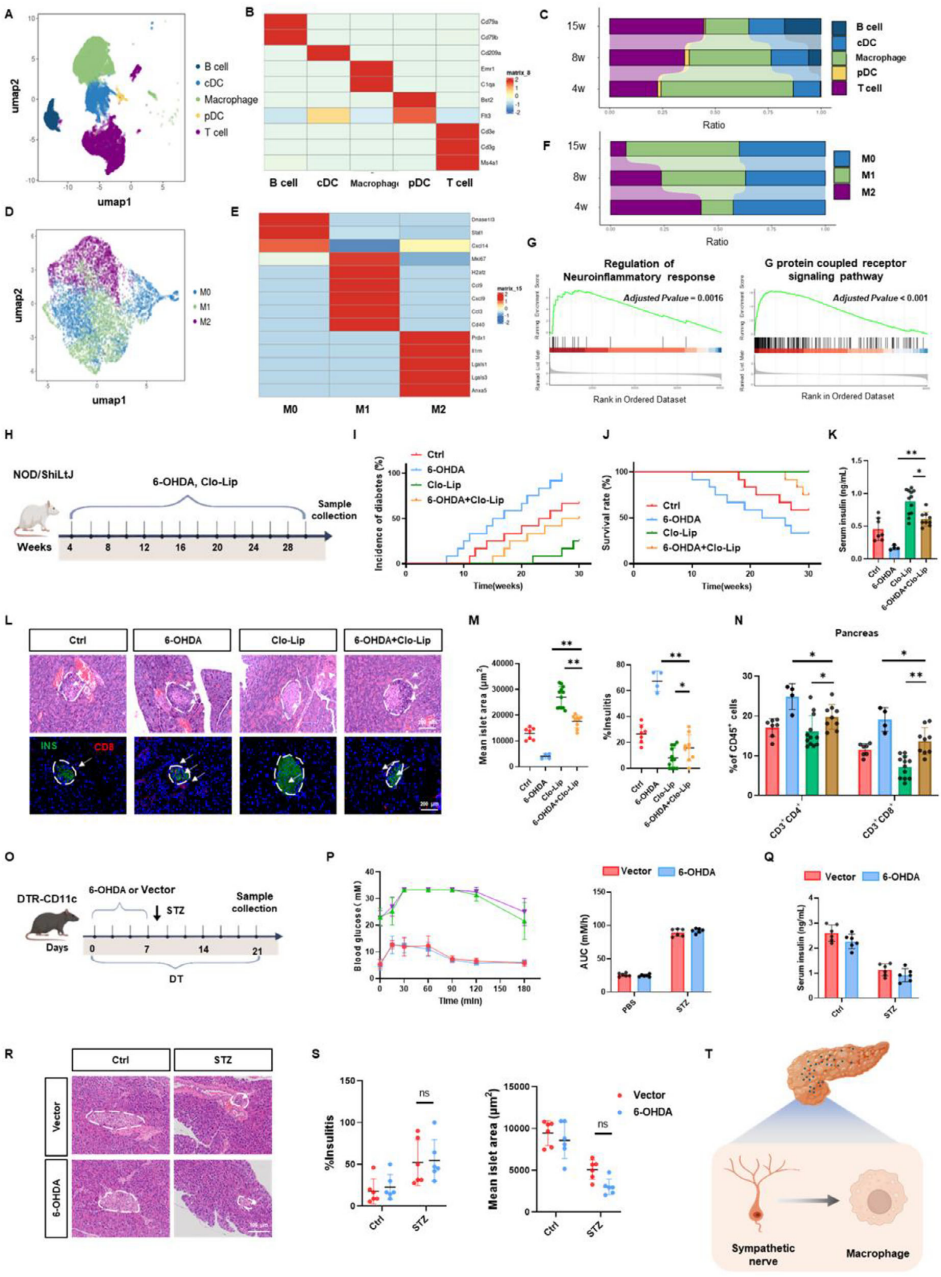
Macrophages within pancreatic islets sense to sympathetic nerve signals throughout the progression of T1D. A) UMAP plot illustrating five distinct cell clusters within the islet immune cell population of NOD mice. B) Heatmap showing the average expression levels of known markers across the five identified cell types. Color intensity indicates the proportion of cells expressing specific genes within each cell type. C) Bar chart illustrating the proportions of the five cell clusters in young NOD mice. D) UMAP plot displaying three distinct cell clusters within the pancreatic macrophage population of NOD mice. E) Heatmap showing the average expression levels of known markers across the three macrophage subpopulations. F) Bar chart illustrating the proportions of the three macrophage clusters in NOD mice of different ages. G) Gene set enrichment analysis (GSEA) of differentially expressed genes in macrophages from NOD mice at different ages. H) Schematic diagram of the mouse experiment. NOD mice were divided into four groups, receiving either saline, 6‐OHDA, clodronate liposomes, or a combination of 6‐OHDA and clodronate liposomes (*n* = 12). I) Incidence of T1D. J) Survival rates. K) Plasma insulin levels. L) Representative H&E staining and immunofluorescence images showing INS and CD8 expression in pancreatic tissues. M) Quantification of average islet area and insulitis scores. N) Flow cytometry analysis of immune cell populations in the spleen, peripheral blood, and pancreatic tissues, showing the proportions of CD3^+^CD4^+^, CD3^+^CD8^+^, F4/80^+^CD11c^+^, and F4/80^+^CD206^+^ cells. O) Flowchart of the experimental design. CD11c‐DTR mice were divided into four groups, receiving intraperitoneal injections of either STZ or citrate buffer, combined with physiological saline containing 0.1% L‐ascorbic acid or 6‐OHDA treatments. P) OGTT on day 21. Q) Plasma insulin levels. R, S) Representative H&E staining images of pancreatic tissues, including quantification of pancreatic inflammation and average islet size. T) Schematic summary of the overall experimental concept and key findings. The data were compiled from at least three independent experiments and are presented as the means ± SEMs. ^*^ *p* < 0.05, ^**^ *p* < 0.01, ^***^ *p* < 0.001 by Student's unpaired two‐tailed *t*‐test or one‐way analysis of variance (ANOVA) with Tukey's post hoc comparison.

Given the autoimmune nature of T1D, we hypothesized that the loss of islet sympathetic nerves during T1D pathogenesis remodels immune cell functions, thereby contributing to disease progression. To investigate this, we analyzed single‐cell data from islet immune cells of NOD mice at various ages. After quality control, dimensionality reduction, and cell annotation, these immune cells were categorized into B cells, T cells, DCs, and macrophage subsets (**Figure** **3**A,B). Over time, the proportions of T and B cells increased, while macrophages predominated in the early stages (Figure 3C). Notably, the proportion of macrophages, particularly proinflammatory macrophages, exhibited significant changes following sympathetic activity inhibition. Consequently, we focused on the role of macrophages in the context of sympathetic nerve loss within pancreatic islets. Detailed analysis of macrophage subsets revealed a gradual increase in proinflammatory macrophages with age in NOD mice, accompanied by a corresponding decline in anti‐inflammatory macrophages (Figure 3D–F). Differential gene expression analysis and Gene Set Enrichment Analysis (GSEA) indicated that the differentially expressed genes were enriched in the regulation of neuroinflammatory response and G protein‐coupled receptor signaling pathways (Figure 3G). Furthermore, 3D immunofluorescence staining demonstrated that macrophages in NOD and STZ mice were in closer proximity to sympathetic nerves, suggesting a strong association with neuronal signals (Figure S6, Supporting Information).

To further clarify this relationship in an autoimmune context, we performed additional experiments in NOD mice using clodronate liposomes (to deplete macrophages), 6‐OHDA (to ablate sympathetic nerves), or the combination of both treatments (Figure 3H). Flow cytometry and immunofluorescence analyses confirmed that clodronate liposome injection reduced F4/80⁺ cells in pancreatic tissues by more than 90%, accompanied by a marked decrease in islet macrophages (Figure S7A,B, Supporting Information). The results showed that sympathetic denervation alone accelerated T1D progression, as evidenced by increased diabetes incidence and mortality, reduced serum insulin levels, enhanced immune cell infiltration, and decreased islet area (Figure 3I–N). In contrast, macrophage depletion alone significantly delayed the onset of diabetes. Compared with 6‐OHDA treatment alone, the combination of macrophage depletion and sympathetic denervation provided partial protection, although the protective effect was less pronounced than that achieved by macrophage depletion alone (Figure 3I–N).

To further validate the involvement of macrophages in T1D in response to sympathetic nerve signaling, we employed a multi‐step experimental approach. First, CD11c‐DTR mice were treated with diphtheria toxin (DT) to achieve selective macrophage depletion.^[^ ^22^ ^]^ Flow cytometry and immunofluorescence analyses confirmed that DT injection reduced CD11c^+^ cells in pancreatic tissues by more than 90%, accompanied by a marked decrease in islet macrophages (Figure S7C,D, Supporting Information). Subsequently, we induced sympathetic nerve depletion in these macrophage‐depleted mice through 6‐OHDA treatment. Finally, we established the T1D model by administering STZ to these animals (Figure 3O). Body weight decreased in all groups, likely due to continuous DT administration; however, 6‐OHDA did not significantly elevate blood glucose levels in CD11c‐DTR mice (Figure S7E,F, Supporting Information). Although glucose tolerance and serum insulin levels showed a downward trend in the 6‐OHDA group, these differences were not statistically significant (Figure 3P,Q). Histological analysis, including CD8 immunofluorescence, revealed no significant increase in islet immune cell infiltration or reduction in islet area in the 6‐OHDA group (Figure 3R,S; Figure S7H, Supporting Information). Additionally, 6‐OHDA treatment did not affect islet single‐cell apoptosis in CD11c‐DTR mice (Figure S7G, Supporting Information). Flow cytometry analysis of pancreatic tissue and whole blood showed no significant changes in immune cell subsets after 6‐OHDA treatment, although a notable increase in CD8^+^ T cells was observed in the spleen (Figure S7I, Supporting Information).

In conclusion, these findings suggest that macrophages within pancreatic islets respond to sympathetic nerve signals during the development of T1D (Figure 3T).

### 2.4 CARD9 is a Regulator for Macrophage Conveying of β2‐AR Signaling

**Figure 4:**
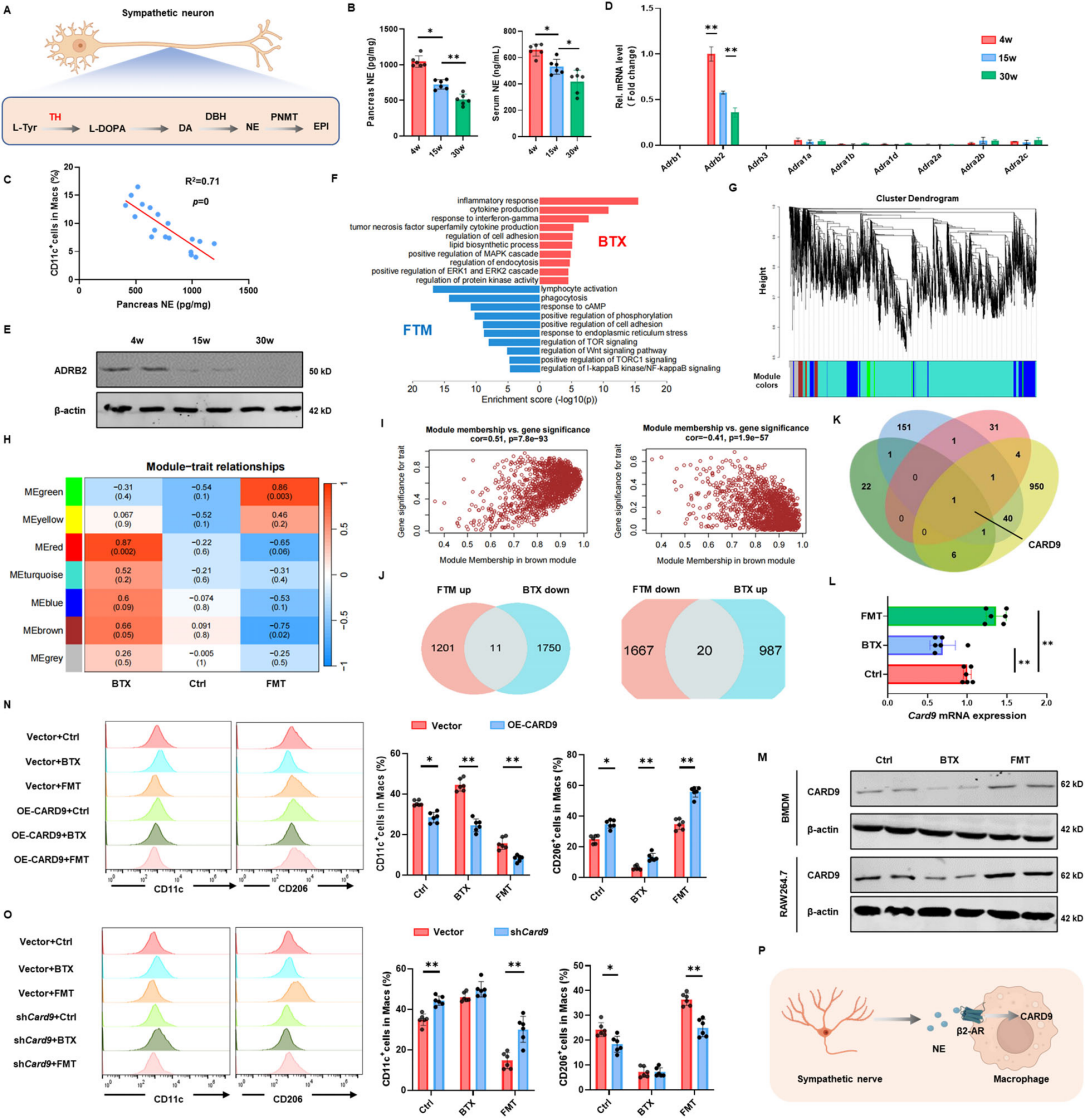
CARD9 is a regulator for macrophage conveying of β2‐AR signaling. A) Flowchart illustrating the synthesis of neurotransmitters by sympathetic neurons. B) NE levels in the serum and pancreatic tissues of NOD mice at different ages. C) Scatter plot showing the correlation between NE levels and CD11c^+^ macrophage populations in the serum of NOD mice. D) Relative transcription levels of NE receptors in pancreatic islet macrophages of NOD mice at different ages. E) Western blot analysis of ADRB2 (β2‐adrenergic receptor) expression in pancreatic islet macrophages of NOD mice at different ages. F) Functional enrichment analysis of differentially expressed genes in BMDMs treated with β2‐AR agonist formoterol (FMT) or β2‐AR inhibitor butoxamine (BTX) compared to controls. G) Gene module clustering dendrogram from weighted gene co‐expression network analysis (WGCNA), illustrating modules formed based on gene co‐expression patterns. H) Module‐trait correlation heatmap from WGCNA, showing the associations between gene modules and treatment. I) Module eigengene‐trait correlation plots from WGCNA, illustrating the relationships between module eigengenes and treatments (FMT and BTX). J) Venn diagrams displaying macrophage activation‐related genes that are inversely regulated by FMT and BTX treatments. K) Venn diagrams displaying genes regulated by macrophages, those co‐regulated by β2‐AR, genes within the “brown” module, and differential gene sets of macrophage subsets in NOD mice. L) qPCR analysis of CARD9 expression levels in BMDMs treated with BTX and FMT. M) Western blot analysis of CARD9 protein expression in BMDMs and RAW264.7 following BTX and FMT treatment. N) Functional analysis of macrophages stably transfected with CARD9 overexpression plasmids. The levels of CD11c and CD206 were measured under the F4/80^+^ gate after exogenous administration of FMT and BTX. O) Functional analysis of macrophages with stable CARD9 knockdown. The levels of CD11c and CD206 were measured under the F4/80^+^ gate following treatment with FMT and BTX. P) Schematic summary of the overall experimental concept and key findings. The data were compiled from at least three independent experiments and are presented as the means ± SEMs. ^*^ *p* < 0.05, ^**^ *p* < 0.01, ^***^ *p* < 0.001 by Student's unpaired two‐tailed *t*‐test or one‐way analysis of variance (ANOVA) with Tukey's post hoc comparison.

The interaction between sympathetic nerves and target cells is primarily mediated by neurotransmitters. To investigate this, we analyzed the levels of NE, epinephrine (EPI), and DA in pancreatic tissue and serum from two T1D mouse models (**Figure** **4**A). The results showed that all three neurotransmitters were associated with a reduction in T1D progression, with NE exhibiting the most pronounced decrease (Figure 4B; Figure S8A–E, Supporting Information). Notably, NE levels were significantly inversely correlated with the proportion of pancreatic CD11c^+^ macrophages (Figure 4C), prompting us to focus on the role of NE in subsequent studies.

NE exerts its effects on target cells through interactions with NE receptors. Among the nine known adrenergic receptor subtypes, pancreatic islet macrophages predominantly expressed *β2‐adrenergic receptor* (*Adrb2*, β2‐AR), with expression levels exhibiting a progressive decline correlating with the duration of T1D progression (Figure 4D; Figure S8F, Supporting Information). Next, we investigated the impact of β2‐AR activation and inhibition on macrophage polarization. Flow cytometry analysis further revealed that BTX (a β2‐AR antagonist) increased the proportion of CD11c^+^ macrophages, whereas FMT (a β2‐AR agonist) significantly enhanced CD206^+^ macrophage populations (Figure S9A–D, Supporting Information). Treatment with BTX significantly increased *IL‐1β* levels in BMDMs, while decreasing *IL‐10* levels. In contrast, FMT exerted the opposite effects (Figure S9E,F, Supporting Information).

To elucidate the mechanisms underlying the effects of β2‐AR on macrophages, we performed RNA‐seq on BMDMs treated with FMT and BTX, respectively. Functional enrichment analysis indicated that FMT significantly upregulated pathways related to lymphocyte activation and cAMP signaling, while downregulating immune response, cytokine production, and IFN‐γ signaling pathways (Figure 4F). Additionally, we employed the Weighted Gene Co‐expression Network Analysis (WGCNA) program to cluster the samples (Figure 4G; Figure S10A,B, Supporting Information). Through dynamic tree cutting and computational analysis, seven gene modules were identified. Correlation analysis between these modules and the treatments generated a correlation heatmap (Figure 4H). Among these co‐expression modules, the “brown” module displayed inverse correlation patterns between receptor activation and inhibition conditions (Figure 4I), highlighting its role as a key regulatory module. Overlap analysis of differential gene sets identified 11 genes upregulated by FMT and downregulated by BTX, and 20 genes downregulated by FMT and upregulated by BTX (Figure 4J). To identify key factors, we compared gene sets for macrophage activation, those co‐regulated by β2‐AR, genes within the “brown” module, and differential gene sets of macrophage subsets in NOD mice. CARD9 emerged as a potential key factor in NE signal transduction (Figure 4K). Western blot and qPCR analysis confirmed that CARD9 expression increased with FMT and decreased with BTX (Figure 4L,M). Moreover, CARD9 expression was lower in islet macrophages from STZ‐induced T1D mice and inversely correlated with NOD mouse age (Figure S10C,D, Supporting Information).

To validate the role of CARD9 in NE signal transduction, we stimulated CARD9 knockdown or overexpressed macrophages with BTX or FMT, respectively (Figure S10E,F, Supporting Information). CARD9 overexpression reduced the increase in CD11c^+^ macrophages induced by BTX, while CARD9 knockdown weakened this effect (Figure 4N). Similarly, the increase in CD206^+^ macrophages induced by FMT was mitigated by CARD9 knockdown and amplified by CARD9 overexpression (Figure 4O). These results suggest that CARD9 acts as a downstream effector of β2‐AR signaling in regulating macrophage activation (Figure 4P).

### 2.5 CARD9 Deficiency Exacerbates Sympathetic Nerve Loss and Promotes Ferroptosis

**Figure 5:**
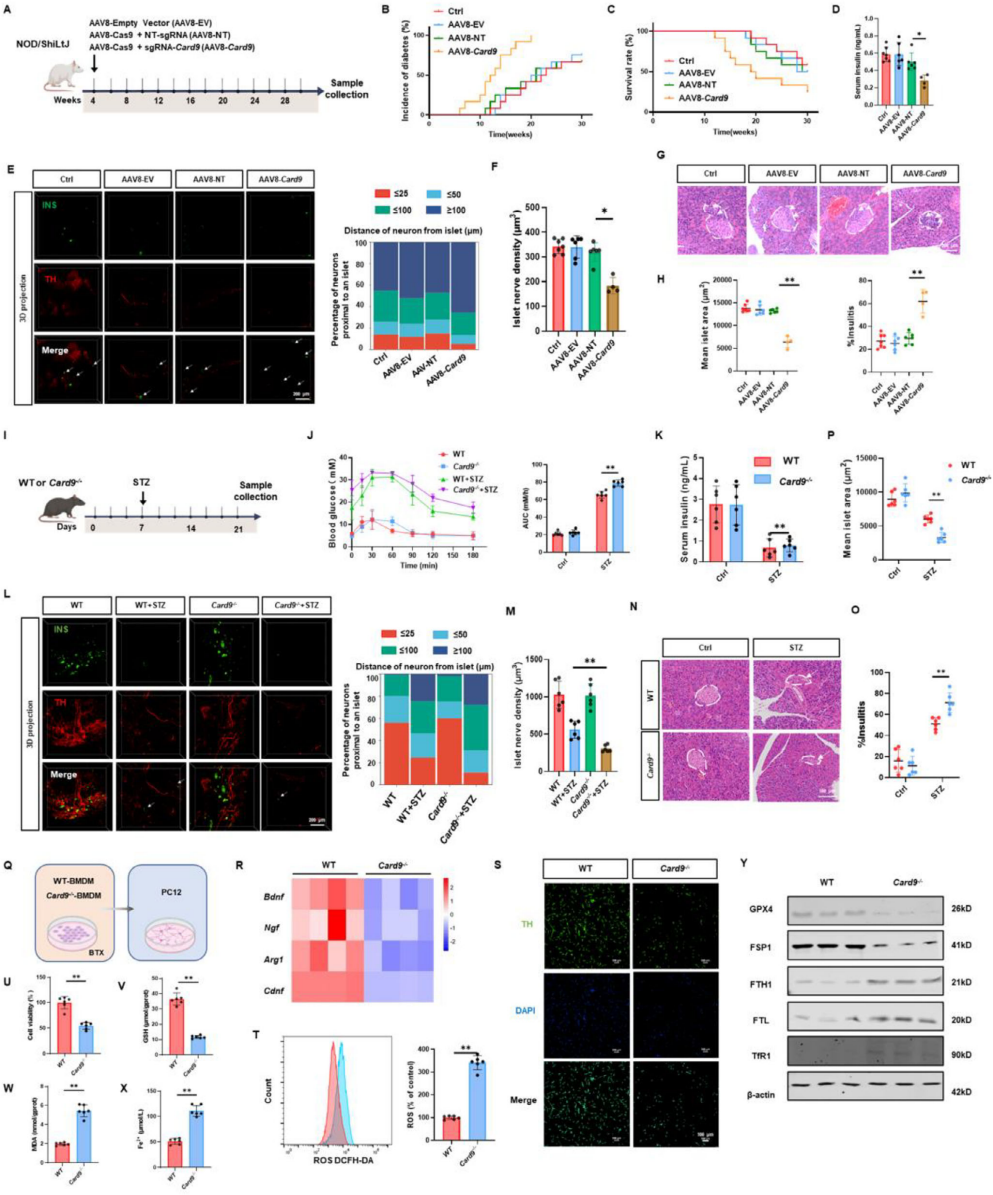
CARD9 deficiency exacerbates sympathetic nerve loss and promotes ferroptosis. A) Schematic diagram of the in vivo experimental design. NOD mice were divided into four groups: AAV8‐Empty Vector (AAV8‐EV), AAV8‐Cas9 with non‐targeting sgRNA (AAV8‐NT), AAV8‐Cas9 with sgRNA‐Card9 (AAV8‐Card9), or saline injection (*n* = 12). B) Incidence of T1D. C) Survival rates. D) Plasma insulin levels. E) Whole‐tissue immunofluorescence imaging of pancreatic tissues from NOD mice at 30 weeks. Insulin is shown in green and TH in red. Sympathetic nerve fiber–islet distances were analyzed and quantified using Imaris software. F) Quantification of islet sympathetic nerve fiber density in NOD mice at 30 weeks. G) Representative H&E staining. H) Quantitative analysis of average islet area and insulitis among different groups. I) Flowchart of the in vivo mouse experiment. WT and *Card9* ^−/−^ mice were divided into four groups, receiving intraperitoneal injections of either citrate buffer or STZ to induce diabetes. J) OGTT on day 21. K) Plasma insulin levels. L) Whole‐tissue immunofluorescence images of pancreatic tissues, with insulin labeled in green and TH in red. M) Quantification of islet sympathetic nerve fiber density using Imaris software. N) Representative H&E staining images of pancreatic tissues. O, P) Quantitative analysis of average pancreatic islet area and insulitis. Q) Schematic overview of the in vitro cell‑based experimental workflow. R) Heatmap depicting the secretion levels of neurotrophic factors, including brain‑derived neurotrophic factor (BDNF), nerve growth factor (NGF), arginase 1 (ARG1), and cerebral dopamine neurotrophic factor (CDNF), in BTX‑treated WT and Card9^−/−^ bone marrow–derived macrophages (BMDMs). S) Representative immunofluorescence images of PC12 cells after co‑culture with BTX‑treated WT or Card9^−/−^ BMDMs. T–X) Quantitative analyses in PC12 cells: reactive oxygen species (ROS) detected by DCFH‑DA fluorescence (T), cell viability (U), intracellular glutathione (GSH) levels (V), malondialdehyde (MDA) levels (W), and ferrous iron (Fe^2+^) accumulation (X). Y) Western blot analysis of proteins related to ferroptosis: glutathione peroxidase 4 (GPX4), ferroptosis suppressor protein 1 (FSP1), ferritin heavy chain 1 (FTH1), ferritin light chain (FTL), and transferrin receptor 1 (TfR1) in PC12 cells. The data were compiled from at least three independent experiments and are presented as the means ± SEMs. ^*^ *p* < 0.05, ^**^ *p* < 0.01, ^***^ *p* < 0.001 by Student's unpaired two‐tailed *t*‐test or one‐way analysis of variance (ANOVA) with Tukey's post hoc comparison.

To investigate the role of CARD9 in pancreatic neuroimmune regulation associated with T1D, we established an AAV‐mediated, pancreas‐specific *Card9* silencing strategy in NOD mice. Mice were injected via the pancreatic duct with AAV8‐Empty Vector (AAV8‐EV), AAV8‐Cas9 with non‐targeting sgRNA (AAV8‐NT), AAV8‐Cas9 with sgRNA‐*Card9* (AAV8‐*Card9*), or saline (**Figure** **5**A). The AAV8 vectors, exhibiting high pancreatic tropism, carried sg*Card9* under the control of the F4/80 promoter to selectively suppress CARD9 expression in pancreatic macrophages. The efficiency of CARD9 knockdown was validated at weeks 5, 15, and 30 (Figure S11A, Supporting Information). *Card9* silencing markedly increased diabetes incidence and reduced survival, accompanied by decreased insulin secretion (Figure 5B–D). Moreover, CARD9 deficiency led to a significant reduction in TH^+^ neuronal numbers, an increased distance between islets and sympathetic nerve terminals, and a pronounced decrease in intra‐islet sympathetic nerve fiber density (Figure 5E,F). Histological evaluation revealed enhanced immune cell infiltration and reduced islet size (Figure 5G,H; Figure S12A, Supporting Information). Flow cytometry further confirmed that CARD9 knockdown promoted the accumulation of CD11c^+^ cells and CD8^+^ T cells within pancreatic tissues (Figure 2N; Figure S12B, Supporting Information).

Furthermore, we generated *Card9* ^−/−^ mice and validated their genetic and protein profiles (Figure S11B, Supporting Information). Both WT and *Card9* ^−/−^ mice were subjected to STZ‐induced T1D for subsequent analysis (Figure 5I). *Card9* ^−/−^ mice exhibited earlier disease onset, significant body weight loss, and elevated blood glucose levels (Figure S13A,B, Supporting Information). OGTT revealed impaired glucose tolerance, and ELISA demonstrated reduced serum insulin levels in *Card9* ^−/−^ mice (Figure 5J,K). Pancreatic tissue imaging showed that *Card9* deficiency led to exacerbated loss of pancreatic sympathetic nerves, accompanied by significant downregulation of *Tubb3* and *Gap43* gene expression (Figure 5L,M; Figure S13C, Supporting Information). Furthermore, *Card9* ^−/−^ mice displayed increased immune cell infiltration in pancreatic islets and a reduction in islet area (Figure 5N–P). *Card9* deficiency was also associated with elevated pancreatic tissue apoptosis, along with increased infiltration of CD11c^+^ macrophages and CD8^+^ T cells (Figure S13D–F, Supporting Information). Notably, *Card9* deficiency alone did not induce hyperglycemia in the absence of STZ treatment. These findings suggest that *Card9* deficiency exacerbates STZ‐induced T1D progression with worsening sympathetic nerve loss.

To assess the role of β2‐AR‐CARD9 in macrophage‐sympathetic cell communication in T1D, we isolated BMDMs from both WT and *Card9* ^−/−^ mice and stimulated them with BTX and subsequently co‐cultured them with PC12 cells (Figure 5Q). *Card9* knockout resulted in decreased secretion levels of neurotrophic factors and anti‐inflammatory factors, including *Bdnf, Ngf, Arg1*, and *Cdnf* in BMDMs (Figure 5R). Additionally, BTX‐induced increases in CD11c^+^ macrophages were amplified by *Card9* deficiency (Figure S13G, Supporting Information).

Ferroptosis plays a pivotal role in both the onset and progression of T1D. Under hyperglycemic conditions, oxidative stress and disrupted iron homeostasis drive the buildup of lethal lipid peroxides, triggering ferroptosis in pancreatic β‐cells and in neurons. At the same time, pro‐inflammatory cytokines such as TNF‐α and IL‐1β exacerbate intracellular oxidative stress and lipid peroxidation, further promoting ferroptosis. By contrast, neurotrophic factors enhance antioxidant defenses and suppress lipid peroxidation, thereby protecting neurons from ferroptosis.^[^ ^23^, ^24^, ^25^ ^]^ Co‐culture with *Card9*‐deficient macrophages significantly increased ROS production, shortened sympathetic neurites, and decreased TH expression in PC12 cells (Figure 5T,U). The resulting surge in labile iron interacted with ROS to form lipid radicals, perpetuating peroxidation.^[^ ^26^ ^]^ Furthermore, co‑culture with CARD9‑deficient macrophages produced a marked decrease in glutathione (GSH) levels and a concomitant increase in malondialdehyde (MDA) and ferrous iron (Fe^2+^) in PC12 neurons (Figure 5U–X). Western blot analyses revealed that loss of CARD9 led to downregulation of the ferroptosis inhibitors glutathione peroxidase 4 (GPX4) and ferroptosis suppressor protein 1 (FSP1), alongside upregulation of the iron‑storage proteins ferritin heavy chain 1 (FTH1), ferritin light chain (FTL), and transferrin receptor 1 (TfR1) (Figure 5Y). Together, these findings demonstrate that CARD9 deficiency in macrophages enhances ferroptosis and worsens sympathetic nerve injury.

To control for endogenous macrophage effects, we transferred BMDMs from both WT and *Card9* ^−/−^ mice, with or without BTX stimulation, into CD11c‐DTR mice and subsequently induced T1D with STZ (Figure S14A, Supporting Information). To verify the localization of donor cells, DIR dye tracking and additional GFP‐labeling experiments confirmed that the transferred BMDMs not only accumulated in the pancreas and liver but also successfully repopulated the islet macrophage compartment (Figure S14B,C, Supporting Information). Among the four BMDM groups, those receiving BTX‐stimulated *Card9* ^−/−^ BMDMs exhibited the most pronounced early‐onset hyperglycemia (Figure S14D, Supporting Information). OGTT confirmed lower glucose tolerance, and ELISA demonstrated reduced serum insulin levels in *Card9* ^−/−^ BMDM recipients (Figure S14E,F, Supporting Information). Moreover, *Card9* ^−/−^ BMDMs exacerbated islet immune cell infiltration, reduced islet area, and increased pancreatic tissue apoptosis, with flow cytometry showing elevated CD8^+^ and CD4^+^ T cells in the spleen (Figure S14G–K, Supporting Information). Together, these findings confirm that CARD9 loss in pancreatic macrophages promotes T1D progression and worsens sympathetic nerve injury.

### 2.6 β2‐AR Signaling Regulates Macrophage Activation via the PKA/CREB1/CARD9 Axis

**Figure 6:**
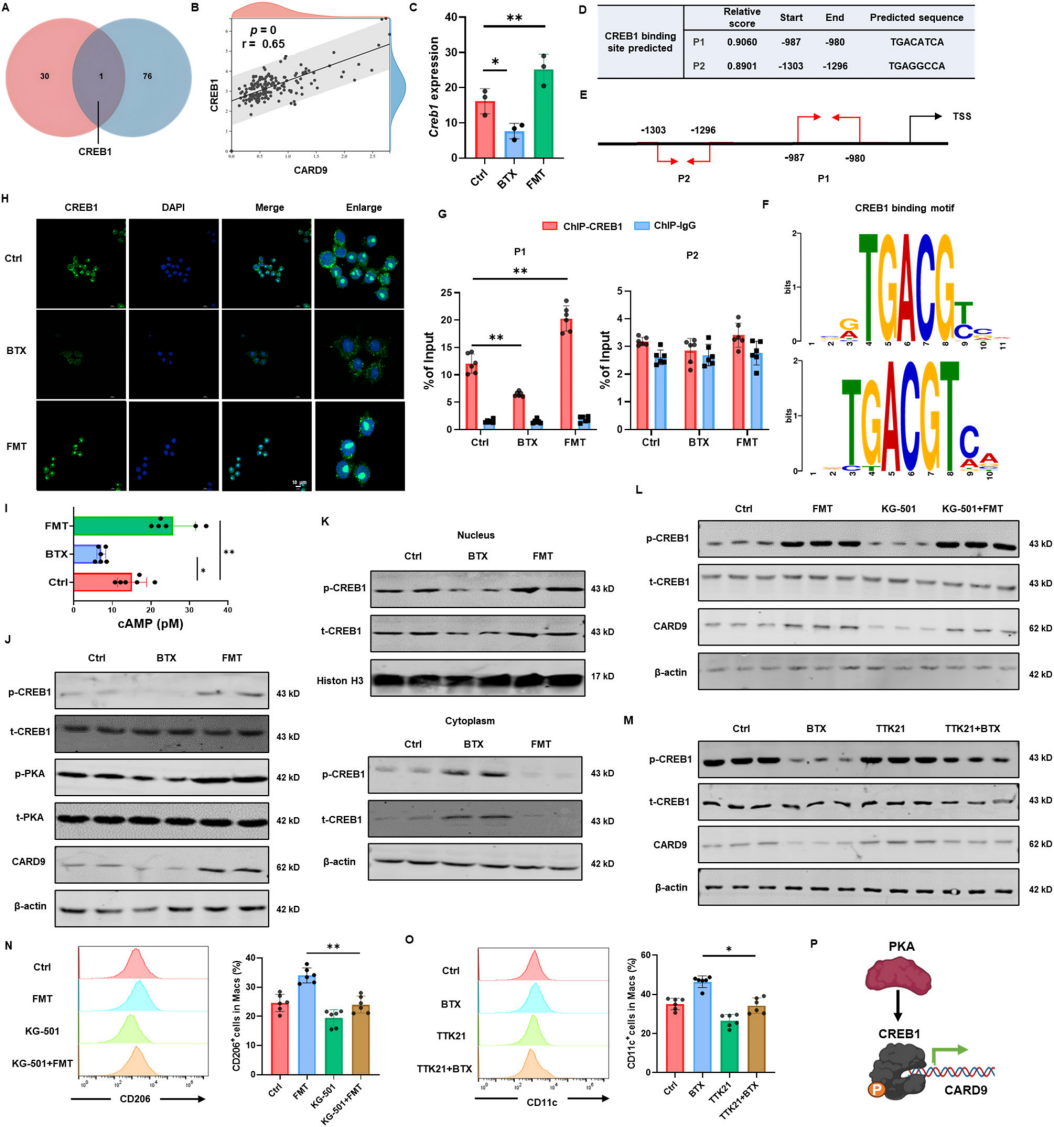
β2‐AR signaling regulates macrophage activation via the PKA/CREB1/CARD9 axis. A) Analysis of the overlap between β2‐AR‐regulated transcription factors and CARD9‐predicted transcription factors. B) Scatter plot showing the correlation between CARD9 and CREB1 expression levels in pancreatic tissues. C) Expression levels of *Creb1* in BMDMs treated with BTX and FMT. D–F) Predicted binding sites of CREB1 within the CARD9 promoter region. G) The binding of CREB1 to *Card9* promoter in BMDM treated with FMT and BTX via chromatin immunoprecipitation (ChIP) assay. H) Immunofluorescence analysis of CREB1 expression in BMDMs following different treatments. I) Levels of cAMP in BMDMs treated with BTX and FMT. J) Western blot analysis of total PKA (t‐PKA), phosphorylated PKA (p‐PKA), total CREB1 (t‐CREB1), phosphorylated CREB1 (p‐CREB1), and CARD9 in BMDMs under various treatment conditions. K) Nuclear and cytoplasmic expression levels of t‐CREB1 and p‐CREB1 in BMDMs. L) Western blot analysis of t‐PKA, p‐PKA, t‐CREB1, p‐CREB1, and CARD9 expression in BMDMs treated with FMT and KG‐501 (a CREB1 inhibitor). M) Western blot analysis of t‐PKA, p‐PKA, t‐CREB1, p‐CREB1, and CARD9 expression in BMDMs treated with BTX and TTK21 (a CREB1 activator). N) CD206^+^ macrophage levels in BMDMs treated with FMT and KG‐501. O) CD11c^+^ macrophage levels in BMDMs treated with BTX and TTK21. P) Schematic summary illustrating the proposed mechanism of NE‐mediated regulation of macrophage activation through the PKA/CREB1/CARD9 signaling axis. The data were compiled from at least three independent experiments and are presented as the means ± SEMs. ^*^ *p* < 0.05, ^**^ *p *< 0.01, ^***^ *p* < 0.001 by Student's unpaired two‐tailed *t*‐test or one‐way analysis of variance (ANOVA) with Tukey's post hoc comparison.

Since β2‐AR modulated *Card9* transcription, we hypothesized that β2‐AR affects the activation of specific transcription factors (TFs). Transcriptomic analysis identified TFs influenced by BTX and FMT, and comparison with predicted TFs for *Card9* highlighted cAMP‐responsive element‐binding protein 1 (CREB1) as a key candidate (**Figure** **6**A). This hypothesis was supported by a significant positive correlation between CREB1 and CARD9 expression in pancreatic tissue (Figure 6B). Further validation through RT‐qPCR confirmed that β2‐AR regulated *Creb1* expression (Figure 6C). Using the JASPAR and AnimalTFDB v4.0 databases, we identified two putative CREB1 binding sites (P1 and P2) in the *Card9* promoter region, both with relative scores above 0.8 (Figure 6D–F). ChIP experiments demonstrated that β2‐AR effectively regulated CREB1 binding to the *Card9* promoter, specifically at the P1 site (Figure 6G).

Transcriptomic functional analysis revealed enrichment in cAMP‐responsive pathways. Further investigation of the canonical cAMP pathway showed that BTX treatment decreased cAMP levels and reduced the phosphorylation of PKA and CREB in macrophages. This reduction was associated with decreased nuclear levels and increased cytoplasmic levels of total CREB1 (t‐CREB1) and phosphorylated CREB1 (p‐CREB1). In contrast, FMT elevated cAMP levels and PKA/CREB1 phosphorylation, increasing nuclear t‐CREB1 and p‐CREB1 while reducing their cytoplasmic levels (Figure 6I–K). Immunofluorescence staining confirmed that BTX inhibited CREB1 nuclear translocation, whereas FMT promoted its nuclear entry (Figure 6H).

To determine whether β2‐AR‐mediated CARD9 regulation depends on CREB1, we treated macrophages with the CREB1 inhibitor KG‐501 and the agonist TTK21. KG‐501 attenuated FMT‐induced CREB1 phosphorylation and CARD9 upregulation, whereas TTK21 counteracted BTX‐mediated suppression of both CREB1 activity and CARD9 expression (Figure 6L,M). KG‐501 also attenuated anti‐inflammatory polarization induced by FMT, whereas TTK21 suppressed pro‐inflammatory polarization triggered by BTX (Figure 6N,O).

Furthermore, to examine whether PKA is required for β2‐AR‐mediated regulation of the CREB1‐CARD9 axis, we treated macrophages with the PKA inhibitor H‐89 and the agonist DC2797. H‐89 attenuated FMT‐induced PKA, CREB1 phosphorylation, and CARD9 upregulation, whereas DC2797 counteracted BTX‐mediated suppression of PKA, CREB1 activity, and CARD9 expression. Flow cytometric quantification further validated these results (Figure S15, Supporting Information). In conclusion, these findings demonstrate that β2‐AR promotes macrophage pro‐inflammatory activation by inhibiting the PKA/CREB1/CARD9 axis (Figure 6P).

### 2.7 β2‐AR Signaling Mediates Macrophage Polarization through CARD9 Regulated Creatine Transport

**Figure 7:**
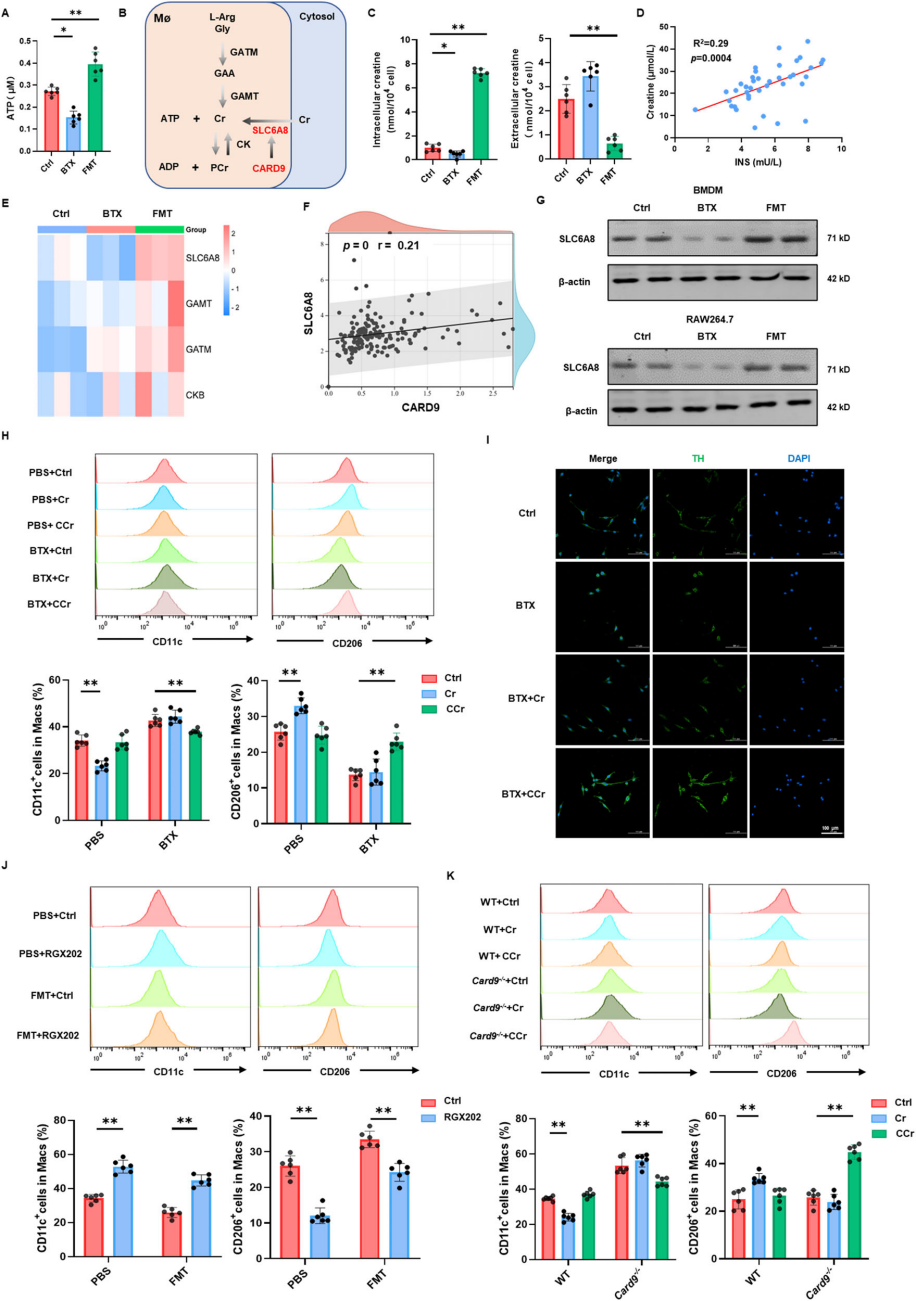
β2‐AR signaling mediates macrophage polarization through CARD9‐regulated creatine transport. A) Intracellular ATP levels in macrophages treated with BTX and FMT. B) Schematic diagram illustrating the process of creatine (Cr) metabolism in macrophages. C) Intracellular and extracellular Cr levels in macrophages following treatment with BTX and FMT. D) A scatter plot illustrating the correlations between insulin and Cr in the plasma of diabetes patients. E) Heatmap showing the relative transcription levels of SLC6A8, GAMT, GATM, and CKB in macrophages treated with BTX and FMT. F) Correlation analysis between CARD9 and SLC6A8 expression levels in pancreatic tissues. G) Western blot analysis of SLC6A8 protein expression in BMDMs and RAW264.7 treated with BTX and FMT. H) Flow cytometry analysis of CD11c and CD206 expression under the F4/80^+^ gating after exogenous administration of Cr and cyclocreatine (CCr) in BMDMs treated with BTX. I) Immunofluorescence analysis of PC12 after co‐culture with exogenous application of Cr and CCr in BMDMs treated with BTX. J) Flow cytometry analysis of CD11c and CD206 expression under the F4/80^+^ gating after exogenous administration of RGX‐202 (a Cr transporter inhibitor) in BMDMs treated with FMT. K) Flow cytometry analysis of CD11c and CD206 expression under the F4/80^+^ gating after exogenous administration of Cr and CCr in WT‐BMDMs and *Card9* ^−/−^‐BMDMs. The data were compiled from at least three independent experiments and are presented as the means ± SEMs. ^*^ *p* < 0.05, ^**^ *p* < 0.01, ^***^ *p* < 0.001 by Student's unpaired two‐tailed *t*‐test or one‐way analysis of variance (ANOVA) with Tukey's post hoc comparison.

Previous research has demonstrated that ATP serves as a substrate to activate the cAMP/PKA signaling pathway.^[^ ^27^ ^]^ In this study, we assessed ATP levels in macrophages and found that BTX significantly inhibited ATP levels, whereas FMT increased ATP levels (**Figure** **7**A). These observations suggest that NE‐β2‐AR may influence macrophage metabolic reprogramming. Building upon earlier studies indicating that CARD9 mediates dendritic cell activation via Cr metabolism, we aimed to investigate whether NE‐β2‐AR modulates macrophage activation through the CARD9‐Cr axis (Figure 7B). The results revealed that BTX significantly reduced intracellular Cr concentrations, while FMT had the opposite effect, increasing intracellular Cr levels (Figure 7C). Interestingly, a significant positive correlation was observed between insulin levels and Cr levels in diabetic patients (Figure 7D). Transcriptomic analysis showed a correlation between SLC6A8 and CARD9, which was corroborated by protein expression data (Figure 7E–G). Moreover, islet macrophages from STZ‐induced T1D mice showed reduced SLC6A8 expression, and SLC6A8 expression negatively correlated with NOD mouse age (Figure S16A,B, Supporting Information).

Given that cyclocreatine (CCr), which is transported into cells independently of SLC6A8, has similar effects to Cr, we treated BMDMs with or without BTX stimulation using Cr or CCr. Cr significantly increased CD206^+^ cells in control BMDMs but had no effect on those treated with BTX. Conversely, CCr reversed the pro‐inflammatory activation induced by BTX, suggesting that CCr that act independently of SLC6A8 mitigate BTX‐induced effects, whereas Cr that relies on SLC6A8 does not (Figure 7H). After adding Cr and CCr to macrophages and coculturing with neural cells, it was found that CCr could promote TH expression in PC12 cells (Figure 7I). Additionally, treatment with the SLC6A8 inhibitor RGX202 reversed the anti‐inflammatory effect of FMT (Figure 7J).

Given the observed decrease in SLC6A8 expression in BMDMs following *Card9* knockout, we hypothesized that β2‐AR regulation of Cr metabolism is CARD9‐dependent. Furthermore, treatment with Cr or CCr showed that Cr significantly increased the proportion of CD206^+^ cells in WT BMDMs but had no effect on *Card9^−/−^* BMDMs. CCr reversed the pro‐inflammatory activation observed in *Card9^−/−^* BMDMs (Figure 7K). In addition, Western blot results with PKA and CREB1 agonists and inhibitors showed that the regulation of SLC6A8 by β2‐AR was CREB1 and CARD9‐dependent (Figure S16C–F, Supporting Information)

In summary, attenuation of β2‐AR signaling downregulates CARD9 expression, thereby promoting the decline of SLC6A8 and creatine deficiency, which promotes pro‐inflammatory macrophage polarization and accelerates the progression of T1D.

## 3 Discussion

Homeostatic imbalance between the nervous and immune systems is closely linked to the onset and progression of various diseases. While previous research has predominantly concentrated on the central nervous system, emerging evidence suggests that dysfunction in the peripheral nervous system plays a pivotal role in disease development.^[^ ^28^, ^29^, ^30^ ^]^ In this study, we conclude that the pancreatic islet sympathetic nervous system regulated pro‐inflammatory macrophage activation, driving T1D onset and progression. Specifically, CARD9 has been identified as a key mediator of β2‐AR signaling from the sympathetic nervous system. Its loss attenuates β2‐AR‐PKA‐CREB1 signaling, reduces intracellular creatine uptake, promotes pro‐inflammatory macrophage polarization, and accelerates the onset of T1D. Moreover, CARD9 reduction in macrophages affects sympathetic nerve loss and promotes ferroptosis by modulating inflammatory and neurotrophic factors. These findings put emphasis on the critical interplay between sympathetic nerves and macrophages in T1D and neuropathy, while also demonstrating that targeting the peripheral sympathetic nervous system can modulate the pancreatic islet immune microenvironment, thereby offering potential therapeutic benefits.

A distinctive and persistent loss of sympathetic neurons was observed specifically within islets in the early stages of T1D, while those in the exocrine pancreas surrounding the islets were well‐preserved. This finding diverges from conventional understanding, as diabetic autonomic and somatosensory neuropathies usually develop over months or years, whereas sympathetic innervation loss in the islets occurs early in T1D.^[^ ^31^, ^32^ ^]^ Significantly, early sympathetic islet neuropathy occurs before overt hyperglycemia. In the rat insulin promoter‐glycoprotein (RIP‐GP) transgenic model, TH^+^ fibers were markedly reduced two days prior to blood glucose elevation.^[^ ^33^ ^]^ Similarly, our data demonstrated that this denervation was not a secondary consequence of hyperglycemia or hypoinsulinemia. Nonautoimmune hyperglycemic models (*db/db* and high‐fat diet–induced mice) displayed preserved or even slightly increased islet innervation. Moreover, insulin glargine supplementation normalized blood glucose in STZ‐induced mice but failed to restore islet sympathetic fibers. We next investigated how sympathetic inhibition influenced T1D onset by applying two approaches: chemical ablation with 6‐OHDA and surgical removal via intraperitoneal CGX. 6‐OHDA is a well‐established neurotoxin that selectively ablates sympathetic nerves, while intraperitoneal CGX surgery eliminates pancreatic sympathetic innervation. Both models revealed a similar pattern: inhibition of sympathetic nerve activity led to increased macrophage and T cell infiltration into the islets, which in turn accelerated the onset of diabetes. Extending this observation, macrophage depletion with clodronate liposomes significantly delayed diabetes onset, while sympathetic ablation alone exacerbated disease. Interestingly, combined macrophage depletion and sympathetic ablation offered partial protection, but the protective effect was weaker than macrophage depletion alone, suggesting that macrophages were critical mediators of sympathetic nerve signaling in islets. In conclusion, early islet sympathetic loss is not merely a bystander phenomenon but represents a pathogenic driver of T1D. Importantly, macrophages emerge as key mediators of sympathetic signaling within islets, providing a mechanistic bridge between neural injury and autoimmune destruction.

CARD9 serves as a central signaling hub in both innate and adaptive immunity.^[^ ^8^, ^9^, ^10^ ^]^ Upon activation, CARD9 selectively forms the CARD9‐BCL10‐MALT1 complex, initiating downstream signaling cascades such as NF‐κB and mitogen‐activated protein kinase (MAPK) pathways.^[^ ^8^ ^]^ In addition, CARD9 promotes IL‐1β secretion via NOD‐, LRR‐ and Pyrin domain‐containing protein 3 (NLRP3) inflammasome activation.^[^ ^34^ ^]^ However, studies by Milton et al. and Xu et al. have presented contrasting findings, demonstrating that CARD9 negatively regulates IL‐1β production in macrophages by inhibiting NLRP3 activation and downregulating IL‐1β secretion through SYK and caspase‐8 modulation.^[^ ^10^, ^35^ ^]^ Our previous research uncovered an additional role of CARD9 beyond its known immune signaling functions. In pancreatic cancer, CARD9 regulated dendritic cell activity by modulating Cr transport through the SLC6A8 transporter.^[^ ^36^ ^]^ This metabolic regulatory function appears to extend to macrophages in T1D. Our findings indicated that β2‐AR signaling activated the PKA‐CREB1 pathway, which subsequently upregulated CARD9. CARD9 then supported macrophage metabolic reprogramming by enhancing creatine uptake and promoting an anti‐inflammatory phenotype. To further substantiate this neuroimmune‐metabolic axis, we established an AAV‐mediated, pancreatic macrophage‐specific *Card9* silencing strategy in NOD mice, as well as generated *Card9* ^−/−^ mice subjected to STZ‐induced T1D. Both models consistently demonstrated that CARD9 deficiency accelerated diabetes onset and markedly worsened sympathetic nerve loss. Moreover, adoptive transfer of BTX‐treated CARD9‐deficient BMDMs into STZ‐induced T1D mice, following endogenous macrophage depletion, resulted in more severe disease outcomes. Notably, therapeutic restoration of sympathetic signaling using the β2‐AR agonist FMT reinstated CARD9 expression, preserved creatine metabolism, maintained anti‐inflammatory macrophage polarization, and protected sympathetic nerves from degeneration. These findings underscore the critical role of CARD9 as a mediator of β2‐AR–driven neuroimmune regulation and highlight its potential as a therapeutic target for mitigating T1D progression and associated neuropathy.

Early alterations in sympathetic signaling are also closely associated with immune cell infiltration. In the RIP‐GP mouse model, sympathetic nerve loss coincided with the infiltration of inflammatory cells. Similarly, in NOD mice, islets with more severe immune infiltration showed greater sympathetic denervation compared to those with milder infiltration.^[^ ^37^, ^38^ ^]^ We found that CARD9‐deficient macrophages exhibited decreased secretion of both anti‐inflammatory cytokines and neurotrophic factors, which may promote sympathetic neuron ferroptosis and contribute to neuropathy. Interestingly, a similar pattern has been observed in the 5xFAD Alzheimer's disease model, where CARD9 deletion led to increased amyloid‐beta (Aβ) deposition, neuronal loss, cognitive decline, and altered microglial activity.^[^ ^13^ ^]^ These observations highlight CARD9 as a potential therapeutic target for both T1D‐associated neuropathy and neurodegenerative diseases.

## 4 Conclusion

In conclusion, we identify CARD9 as a critical mediator linking β2‐AR signaling in macrophages to creatine metabolism, immune activation, and neuronal integrity. Loss of sympathetic input impairs β2‐AR‐PKA‐CREB1‐CARD9 signaling, reduces Cr uptake, and promotes a shift toward pro‐inflammatory macrophage polarization. This shift not only accelerates β‐cell autoimmunity but also drives sympathetic axon ferroptosis due to diminished neurotrophic and anti‐inflammatory factor secretion. Importantly, restoring sympathetic signaling with a β2‐AR agonist FMT rescues CARD9 expression, enhances Cr uptake, and maintains anti‐inflammatory macrophage polarization. Collectively, these findings elucidate novel mechanisms underlying neuroimmune network dysregulation in the pathogenesis of T1D, highlighting potential neuroprotective strategies for the development of new therapeutic interventions.

## 5 Experimental Section

### Clinical Information

Between January 2025 and April 2025, patients diagnosed with diabetes were enrolled at Union Hospital, Tongji Medical College, Huazhong University of Science and Technology, Wuhan, China. No organs were obtained from executed prisoners in this study. Serum samples were collected from all participants with informed consent or appropriate authorization (Table S1, Supporting Information). The enrolled patients met all of the following diagnostic criteria: random venous plasma glucose ≥ 11.1 mmol L^−1^, glycated hemoglobin (HbA1c) ≥ 6.5%, and the presence of islet autoantibodies (IAA, among others). The study was approved by the Ethics Committee of Union Hospital, Tongji Medical College, Huazhong University of Science and Technology (UHCT‐IEC‐SOP‐016‐03‐01) and conducted in accordance with the principles of the Declaration of Helsinki. Informed consent was obtained from all participants.

### Animal Information

All surgical and experimental procedures involving mice were ethically approved by the Institutional Animal Care and Use Committee of Tongji Medical College (2024 IACUC Number: 4301). The mice were housed in a 12‐h light/dark cycle at 23 ± 2 °C, with free access to food and water, and were maintained under specific pathogen‐free conditions. Male wild‐type (WT) C57BL/6J mice were purchased from Beijing Vital River Laboratory Animal Technology Co., Ltd. (Beijing, China), while *Card9^−/−^* mice and CD11c diphtheria toxin receptor (DTR) mice with a C57BL/6J background were purchased from Cyanen Biosciences Inc. (Suzhou, China) and Shulaibao Biosciences Inc. (Wuhan, China), respectively. These mice were generated and described in the previous studies. NOD/HiLtJ mice were purchased from Bainet Biotechnology Co. (Wuhan, China). All animal experiments were conducted using a blinded method, without statistical calculations to pre‐determine the sample size for in vivo experiments.

### Animal Experiments

The animal experiments were divided into five parts, with each critical experiment independently repeated three times in separate cohorts to ensure reproducibility. Group sizes for each experiment are indicated below:

First, sympathetic nerve changes were monitored in female NOD mice (*n* = 10) across different disease stages and ages. T1D was also induced in 8‐week‐old male C57BL/6J mice (*n* = 6) by intraperitoneal injection of streptozotocin (STZ; 100 mg kg^−1^ day^−1^ for 3 days; S0130, Sigma–Aldrich, USA), using citrate buffer as the control. Only mice with fasting blood glucose (FBG) ≥ 11.1 mm were classified as diabetic and included in the analysis of sympathetic nerve changes. In addition, male NOD mice (*n* = 12) and female mice (*n* = 8) in the STZ‐induced T1D model were included in validation experiments to assess potential sex‐dependent effects.

Second, 12‐week‐old *db/db* mice (*n* = 8) and high‐fat diet–induced hyperglycemic mice that had been fed a high‐fat diet for 8 weeks (*n* = 8) were used to determine whether islet sympathetic nerve loss was secondary to hyperglycemia. In STZ‐induced T1D mice, long‐acting insulin (insulin glargine; 5 U kg^−1^ day^−1^; 00088221905, Sanofi, France) was administered daily (*n* = 8). These mice were used to determine whether islet sympathetic nerve loss was secondary to hypoinsulinemia.

Third, to investigate the effects of sympathetic nerve inhibition on T1D, female NOD mice (*n* = 8) were treated with 6‐hydroxydopamine (6‐OHDA, 100 mg kg^−1^; H4381, Sigma–Aldrich, USA) or subjected to celiac ganglionectomy (CGX), with STZ‐induced T1D mice (*n* = 6) undergoing the same procedures.

Fourth, to explore the relationship between sympathetic nerves and macrophages in T1D, mice were treated with clodronate liposomes (150 µL per mouse by intraductal injection into the pancreas initially, followed by weekly tail vein injections of 150 µL; CP‐005‐005, LIPOSOMA BV, Netherlands), 6‐OHDA (100 mg kg^−1^), or a combination of both (*n* = 12 per group). In addition, CD11c‐DTR mice (*n* = 6) were depleted of CD11c^+^ macrophages by intraperitoneal injection of diphtheria toxin (DT; 6 ng g^−1^ in 100 µL PBS; D0564, Sigma–Aldrich, USA) every 48 h, followed by STZ‐induced T1D and subsequent 6‐OHDA treatment.

Finally, to investigate the role of Card9 in neuroimmune regulation during T1D, an AAV‐mediated, pancreas‐specific *Card9* silencing strategy was established in NOD mice. Mice were injected via the pancreatic duct with AAV8‐Empty Vector (AAV8‐EV), AAV8‐Cas9 with non‐targeting sgRNA (AAV8‐NT), AAV8‐Cas9 with sgRNA‐*Card9* (AAV8‐*Card9*), or saline (*n* = 12). The AAV8 vectors, which exhibit high pancreatic tropism, carried sgCard9 under the control of the F4/80 promoter to specifically target macrophages. STZ‐induced T1D was also established in WT and *Card9* ^−/−^ mice (*n* = 6). In a separate cohort, CD11c‐DTR mice received DT treatment, followed by STZ and 6‐OHDA administration. For further mechanistic studies, BMDMs from WT or *Card9* ^−/−^ mice, with or without butoxamine (BTX, HY‐119868, MedChemExpress, USA) stimulation, were transplanted into CD11c‐DTR mice (5 × 10^6^ cells per mouse; *n* = 6).

### Chemical Sympathetic Nerve Resection Surgery

Dissolve 6‐OHDA in sterile saline containing 0.1% L‐ascorbic acid (A8100, Solarbio, China) as an antioxidant. Intraperitoneal injection of 6‐OHDA into mice depletes the sympathetic nervous system. Control animals were injected with physiological saline containing 0.1% L‐ascorbic acid.

### Celiac Ganglionectomy (CGX)

Mice underwent surgery under isoflurane anesthesia. A midline laparotomy exposed the abdominal ganglion area, followed by dissection and removal of the abdominal plexus and associated nerves near major blood vessels, including the aorta, abdominal artery, and mesenteric artery. The abdominal incision was closed in two layers with intermittent sutures.^[^ ^21^ ^]^ In sham‐operated mice, the ganglion plexus was exposed but not excised. Post‐surgery, mice were given 1 week to recover.

### Depletion of Pancreatic Macrophages using Clodronate Liposomes and DT

To selectively deplete pancreatic macrophages, mice were treated with clodronate liposomes. Mice were maintained on a standard diet and acclimatized for at least 1 week prior to the experiment. Clodronate liposomes were administered via intraductal injection at a dose of 100 µL per 20 g body weight, while control mice received an equal volume of PBS. Injections were performed 48 and 24 h prior to the experiment to ensure efficient macrophage depletion. Following treatment, mice were sacrificed, and pancreatic tissue was harvested. Single‐cell suspensions were prepared by collagenase IV (17104019, Thermo Fisher Scientific, USA) and trypsin (15090046, Thermo Fisher Scientific, USA) digestion with trypsin, and depletion efficiency was assessed by flow cytometry based on the frequency of CD45^+^F4/80^+^CD11b^+^ macrophages.

For depletion of CD11c^+^ macrophages, DT was dissolved in sterile PBS and administered intraperitoneally at 10 ng g^−1^ body weight, with control mice receiving PBS. DT was injected 24 h before the experiment and again on the day of the experiment to ensure effective depletion. After treatment, mice were sacrificed, and pancreatic tissue was collected. Single‐cell suspensions were generated by collagenase IV and trypsin digestion, and flow cytometry was used to evaluate the proportion of CD45^+^F4/80^+^CD11c^+^ macrophages to confirm depletion efficiency.

### Small Animal Imaging

Biological distribution of injected BMDMs was assessed using DiR labeling (HY‐D1048, MedChemExpress, USA). BMDMs were suspended in 5 µg mL^−1^ DiR buffer and incubated at 37 °C for 20 min to label macrophages, then administered to mice via tail vein injection. Pancreas, lungs, liver, spleen, kidneys, and heart were collected and imaged using the Pear Trilogy System (Odyssey CLX, USA).

### Cell Culture and Transfection

RAW264.7 and PC12 cells were cultured in DMEM (SH30022.01, Cytiva, USA) supplemented with 10% fetal bovine serum (FBS, 26140079, Thermo Fisher Scientific, USA) and 100 U mL^−1^ penicillin‐streptomycin (15140122, Thermo Fisher Scientific, USA). Mouse BMDMs from WT or *Card9^−/−^* mice were cultured in differentiation medium consisting of RPMI 1640 (11875093, Thermo Fisher Scientific, USA) supplemented with 10% FBS and 20 ng mL^−1^ recombinant mouse M‐CSF (HY‐P7085, MedChemExpress, USA). During the 5‐day differentiation period, cells were continuously exposed to β2‐adrenergic receptor agonist formoterol (FMT, HY‐B0010, MedChemExpress, USA) and β2‐adrenergic receptor antagonist butoxamine (BTX, HY‐119868, MedChemExpress, USA). On day 6, differentiated macrophages were stimulated with 100 ng mL^−1^ lipopolysaccharide (LPS, L2880, Sigma–Aldrich, USA) for 4 h for downstream analysis. RAW264.7 murine macrophages and BMDMs were plated in 6‐well culture plates at 1 × 10^6^ cells mL^−1^. Following 24 h of adhesion in complete medium, cells were serum‐starved for 24 h to synchronize cell cycle progression prior to stimulation. For β2‐AR studies, cells were pre‐treated with FMT (100 nm) or BTX (10 µm) for 2 h and then treated with NE (100 nm; HY‐13715, MedChemExpress, USA) for 12 h.^[^ ^39^, ^40^, ^41^ ^]^ The mouse CARD9 expression plasmids and sh*Card9* were obtained from Gene Create (Wuhan, China). Macrophage cells were transfected with the vector or the protein expression plasmids using NeofectTM (NB‐24‐00001, Neofect Biotech, China).

### Lentiviral Transduction of BMDMs with GFP

On day 2 of BMDM differentiation, cells were transduced with GFP‐expressing lentivirus in the presence of polybrene (8 µg mL^−1^; H9268, Sigma–Aldrich, USA) to enhance infection efficiency. The culture plates were centrifuged at 1250 × g for 60 min at 30 °C, followed by incubation at 37 °C for 12 h to facilitate viral entry. After infection, cells were gently washed twice with PBS and cultured in complete medium supplemented with M‐CSF for an additional 48 h to allow GFP expression. GFP fluorescence was examined 48 h post‐transduction using an inverted fluorescence microscope (Olympus, Japan) with excitation at 488 nm. For in vivo tracking, GFP‐labeled BMDMs were administered to mice via tail vein injection. The distribution of GFP^+^ cells in target tissues was assessed by immunofluorescence staining.

### Cell Viability Assay

BMDMs were seeded into 96‐well plates at a density of 1 × 10^4^ cells per well and allowed to adhere overnight. Cells were then treated with NE at concentrations of 10, 100, or 1000 nm for 24 h, while control wells received an equal volume of vehicle. Cell viability was assessed using the CCK‐8 assay (abs580086, Absin, China) according to the manufacturer's instructions. Briefly, 10 µL of CCK‐8 reagent was added to each well, followed by incubation at 37 °C for 1 h. Absorbance was measured at 450 nm using a microplate reader (BioTek Instruments, USA), and cell viability was calculated relative to control wells. All experiments were performed under sterile conditions and independently repeated at least three times to ensure reproducibility.

### Oral Glucose Tolerance Test (OGTT)

After an overnight fast (16 h), mice were administered glucose (HY‐B0389, MedChemExpress, USA) orally by gavage (2 g kg^−1^ body weight, 20% ddH_2_O solution). Blood glucose levels were measured using a handheld glucose meter (Sinocare, China) at 0, 15, 30, 60, 90, 120, and 180 min post‐glucose administration, via tail vein sampling.

### Immunophenotype Analysis

Immune cells from whole blood, pancreas, and spleen were analyzed by flow cytometry (FCM). Pancreatic single‐cell suspensions were prepared by enzymatic digestion with collagenase P (11213857001, Roche, Switzerland), thereby yielding both islet‐ and exocrine‐resident immune cell populations. Spleen cells were prepared by physical dissociation. Whole blood single cells were first treated with red blood cell lysis buffer before antibody incubation. Cell surface antigens were stained for 60 min at 4 °C using fluorescently labeled antibodies targeting specific antigens. 1) Macrophages were stained with FITC‐CD11b (553311, BD Biosciences, USA) and PE‐F4/80 (565410, BD Biosciences, USA). M1 and M2 macrophages in vivo were identified using APC‐CD11c (550261, BD Biosciences, USA) or Alexa 647‐CD206 (565250, BD Biosciences, USA), respectively. M1 or M2 types of BMDM and RAW264.7 in vitro were identified using FITC‐CD11c (561045, BD Biosciences, USA) or Alexa 647‐CD206, respectively. 2) CD8^+^ T cells were stained with APC‐CD3 (561826, BD Biosciences, USA) and PE‐CD8 (568906, BD Biosciences, USA), while CD4^+^ T cells were stained with APC‐CD3 and FITC‐CD4 (561828, BD Biosciences, USA). Flow cytometry was performed using a C6 flow cytometer (BD Accuri, USA), and data were processed using FlowJo 10 (BD Life Sciences, USA).

### Cell Apoptosis Assay

Dispersed pancreatic cells were filtered through a 40 µm mesh and washed with Annexin V binding buffer (42201, Biolegend, USA). After filtration, cells were incubated with FITC‐conjugated membrane‐associated protein V and propidium iodide (640914, Biolegend, USA). Cells positive for membrane‐associated protein V and PI were considered apoptotic. The sample was run on a C6 flow cytometer and analyzed using FlowJo 10.

### Histology and Immunofluorescence

Mice were euthanized, and their pancreas excised and fixed in formalin (01‐337‐234, Thermo Fisher Scientific, USA), then embedded in paraffin and sectioned at a thickness of 5 µm at three depths, with 200 µm intervals between sections. Sections from each depth were stained with H&E and scored for insulitis as previously described. Periinsulitis was defined as a focal aggregation of inflammatory cells at one pole of the islet. Non‐invasive insulitis refers to lesions with clear but usually extensive islet infiltration, occupying less than 50% of the islet area, while invasive insulitis involves more severe infiltration, where lymphoid cells invade the entire islet, mixing with endocrine cells and causing extensive beta cell damage.

For immunofluorescence, sections were stained with primary antibodies, followed by incubation with Alexa Fluor 594‐conjugated anti‐rabbit IgG (8889, CST, USA), Alexa Fluor 488‐conjugated anti‐rabbit IgG (4412S, CST, USA), and Alexa Fluor 488‐conjugated anti‐mouse IgG (4408, CST, USA). DAPI (1:1000 dilution; D9542 Sigma–Aldrich, USA) was used to stain nuclei. Images were acquired with confocal microscopy (LSM780, Germany), and co‐localization analysis was performed using ImageJ (NIH, USA). The primary antibodies used are listed in Table S2 (Supporting Information).

### Whole Tissue Immunostaining and Imaging

Mice were anesthetized and perfused with PBS supplemented with 10 µg mL^−1^ heparin (H3149, Sigma–Aldrich, USA). Pancreatic tissue was dissected, fixed in PBS with 1% paraformaldehyde (PFA, 158127, Sigma–Aldrich, USA) and 10% sucrose (S9378, Sigma–Aldrich, USA), washed with PBS, and dehydrated using a graded methanol series. It was bleached with 5%H_2_O_2_ (H1009, Sigma–Aldrich, USA) and 10 mm ethylenediaminetetraacetic acid (EDTA, pH 8.0, HY‐Y0682R, MedChemExpress, USA), prepared by diluting 1 part 30% H_2_O_2_ in 5 parts 100% methanol (M116114, Aladdin, China), then rehydrated using a reverse methanol gradient. Permeabilization was performed in PBS containing 0.2% Triton X‐100 (T9284, Sigma–Aldrich, USA), 20% dimethyl sulfoxide (DMSO, D8418, Sigma‐Aldrich, USA), and 0.3 m glycine (G8898, Sigma–Aldrich, USA), followed by blocking with PBS containing 0.2% Triton X‐100, 10% DMSO, and 5% donkey serum (017‐000‐121, Jackson Immuno Research, USA). Tissue was incubated with primary antibody diluted in PBS with 0.2% Tween‐20 (P7949, Sigma–Aldrich, USA), 10 µg mL^−1^ heparin, 5% DMSO, and 5% donkey serum, washed with PBS containing 0.2% Tween‐20 and 10 µg mL^−1^ heparin, and incubated with secondary antibody conjugated to Alexa dye (1:500 dilution) in PBS with the same additives. After washing, the immunolabeled pancreas was embedded in 1% agarose prepared in PBS, dehydrated, optically cleared, and imaged using a light microscope. Image stacks from optical imaging were reconstructed using Imaris 9.0 (Bitplane, Switzerland), which quantified distances between insulin signals and tyrosine hydroxylase (TH^+^) nerve fibers with the Spots function.^[^ ^42^ ^]^ Shortest distances from spots to surfaces were measured to generate curves. The primary antibodies used are listed in Table S2 (Supporting Information).

### Islet Isolation

Pancreatic islets were isolated using a standard collagenase/protease digestion method. A 0.5 mg mL^−1^ collagenase/protease solution was incubated at 4 °C to dilate ducts, and the reaction was halted with 10% fetal bovine serum in RPMI 1640. The mixture was washed three times with 0.02% bovine serum albumin (BSA, A7906, Sigma–Aldrich, USA) in 1X Hank's balanced salt solution (Corning). Islets were separated from exocrine tissue using a Histoque‐1077 (10771, Sigma–Aldrich, USA) gradient in serum‐free RPMI 1640. Before experiments, islets were manually selected and cultured overnight at 37 °C.^[^ ^43^ ^]^

### Isolation of Pancreatic Islet Macrophages

Pancreatic islets were first isolated from mice using standard collagenase digestion and density gradient centrifugation. The islets were then dissociated into a single‐cell suspension. Macrophages were purified from this suspension using the MagniSort Mouse F4/80 Positive Selection Kit (8802‐6863‐74, Thermo Fisher Scientific, USA), which utilized a biotinylated anti‐F4/80 antibody and streptavidin‐coated magnetic beads. The cells were incubated with the beads at 4 °C for 15 min in MACS buffer (PBS supplemented with 0.5% BSA and 2 mm EDTA), and then passed through a pre‐washed LS column (130‐042‐401, Miltenyi Biotec, Germany) according to the manufacturer's instructions. Unbound cells were removed in the flow‐through, while F4/80^+^ macrophages were retained and subsequently eluted. The purity of the isolated islet macrophages was routinely assessed by flow cytometry and was typically >90%. These purified macrophages were subsequently used for analysis of adrenergic receptor expression.

### RNA Sequencing

BMDMs were pre‐treated with FMT (100 nm) or BTX (10 µm) for 2 h and then treated with NE (100 nm) for 12 h. RNA purity and integrity were assessed using the MGI system for RNA sequencing. Raw reads were processed for quality control using Fastp (version 0.23.2). Clean reads were mapped using Hisat2 (version 2.2.1), followed by gene quantification with RSEM (version 1.3.1) and differential expression analysis with DESeq2 (version 1.4.5).^[^ ^44^ ^]^

### ATP and Creatine Content Detection

Samples were prepared to detect creatine levels using a creatine assay kit (ab204537, Abcam, UK) and ATP levels using an ATP estimation kit (K354‐100, BioVision Inc., USA), according to each protocol.

### Glutathione (GSH) and Malondialdehyde (MDA) Content Detection

Levels of GSH and MDA were determined using commercial assay kits (S0052, S0131S; Nanjing Jiancheng, China), in accordance with the manufacturer's instructions.

### Ferrous Iron (Fe^2+^) Content Detection

Levels of Fe^2+^ were determined using commercial assay kits (E‐BC‐F101, Elabscience, China), in accordance with the manufacturer's instructions.

### ELISA

Serum and pancreatic levels of NE, DA, EPI, and insulin in mice were quantified using ELISA according to the manufacturer's instructions. Likewise, serum levels of insulin, NGF, BDNF, LIF, and CNTF in patients were measured following the respective protocols. In addition, levels of Arg‐1, IL‐1β, TNF‐α, cAMP, IL‐10, BDNF, NGF, and CNTF in BMDMs were determined by ELISA. Detailed information on the ELISA kits is provided in Table S4 (Supporting Information).

### Chromatin Immunoprecipitation (ChIP)‐qPCR

ChIP detection was performed using the Simple ChIP Kit (9004S, CST, USA). BMDM cells were collected after treatment for 48 h and crosslinked with 1% formaldehyde at room temperature. Immunoprecipitation was carried out by enzymatically cleaving DNA and using specific ChIP‐grade antibodies (4820, CST, USA) targeting CREB1. IgG (2729, CST, USA) was used as an internal control. Relative quantitative analysis was performed by qPCR.

### RT‐qPCR

Total mRNA was extracted using Trizol reagent (15596026, Thermo Fisher Scientific, USA) according to the manufacturer's instructions. cDNA synthesis was performed using the GoScript Reverse Transcription System (A5000, Promega, USA). qPCR was conducted using the GoTaq qPCR Master Mix (A6001, Promega, USA) on the CFX Connect Real‐Time PCR Detection System (Bioer, Hangzhou, China). mRNA levels were normalized to β‐actin levels. The primers used are summarized in Table S3 (Supporting Information).

### Western Blotting

Proteins were separated by sodium dodecyl sulfate polyacrylamide gel electrophoresis (SDS‐PAGE), and separated bands were transferred to a polydifluoroethylene membrane (PI88518, Thermo Fisher Scientific, USA). After blocking with 5% BSA, the membrane was incubated with primary antibody at 4 °C for 12 h, followed by incubation with IRDye 800‐labeled goat anti‐rabbit or anti‐mouse secondary antibodies (5151S, 5257P; CST, USA). Anti‐β‐actin antibodies served as a loading control. Images were captured using the Odyssey imaging system CLx (LI‐COR Biosciences, USA) and quantified through ImageJ. The primary antibodies used are listed in Table S2 (Supporting Information).

### Quantification of Sympathetic Nerve Fiber Density in Pancreatic Islets

3D image analysis was performed using Imaris software (Bitplane, Switzerland). After background subtraction and signal enhancement, TH‐positive sympathetic nerve fibers and terminals were identified and reconstructed using the “Filament Tracer” or “Spots” modules. Sympathetic nerve fiber density within pancreatic islets was defined as the number of nerve terminals per unit volume (fibers/µm^3^). Image acquisition and processing parameters were kept consistent across all experimental groups to ensure data comparability.

### Transcriptome and Serum Protein Analysis of T1D Pancreatic Islet Tissue

RNA sequencing data of pancreatic islet tissue from T1D patients were obtained from GSE181674, and serum samples from T1D patients were obtained from GSE50866. Gene quantification and differential expression analysis were performed using DESeq2 (version 1.4.5). GO enrichment analysis (adjusted for a *p*‐value < 0.01 using Bonferroni correction) and KEGG enrichment analysis were conducted on marker genes in each cluster using the R package clusterProfiler (functional comparisons of Cluster, enrichGO, and enrichKEGG), as well as on differentially expressed genes or proteins across groups.^[^ ^45^ ^]^

### Single‐Cell RNA‐Seq

Single‐cell transcriptome data were analyzed using R (version 4.1.2, 2021‐11‐01). Single‐cell RNA sequencing data of pancreatic islet tissue from T1D patients were obtained from GSE148073, while data for immune cells in NOD mouse pancreatic tissue were retrieved from GSE141784 (GSM4213196 to GSM4213200). Data normalization, clustering, differential gene expression analysis, and visualization were performed using Seurat (version 4.2).^[^ ^46^ ^]^ Cells were clustered with the FindClusters function and visualized using RunUMAP. Specific marker genes for each cell cluster were identified by FindMarkers, while classic marker genes and top differentially expressed genes were used to annotate cell types.

### Statistics

Data analysis was conducted using GraphPad Prism 9 (GraphPad Software, USA). Comparisons between two groups were analyzed with an unpaired two‐tailed Student's t‐test, while time course data comparisons involving two or more groups used two‐way ANOVA. Each “*n*” representing the number of mice is specified in the figure legends. Statistical significance was set at *p* ≤ 0.05, with significance levels defined as ^*^ *p* ≤ 0.05, ^**^ *p* ≤ 0.01, ^***^ *p* ≤ 0.001, ^****^ *p* ≤ 0.0001, and n.s. (not significant). Error bars indicate the standard error of the mean (SEM).

## Conflict of Interest

The authors declare no conflict of interest.

## Author Contributions

H.Y. and S.L. contributed equally to this work. H.Y. did the writing of the original draft, visualization, validation, methodology, investigation, formal analysis, data curation, and conceptualization. S.L. did the writing of the original draft, visualization, validation, methodology, investigation, formal analysis, data curation, conceptualization, and funding acquisition. S.L. did visualization, validation, investigation, formal analysis, data curation, and conceptualization. H.Z., Y.X., W.L., M.W., M.W., M.Z., W.S., X.L., and Y.F. did formal analysis and data curation. M.X. did the writing of the review & editing, supervision, funding acquisition, and conceptualization.

## Supporting information



Supporting Information

Supporting Video 1

Supporting Video 2

## Data Availability

The data that support the findings of this study are available from the corresponding author upon reasonable request.

## References

[1] K. C. Herold , T. Delong , A. L. Perdigoto , N. Biru , T. M. Brusko , L. S. K. Walker , Nat. Rev. Immunol. 2024, 24, 435. 38308004 10.1038/s41577-023-00985-4 PMC7616056

[2] N. Martinez‐Sanchez , O. Sweeney , D. Sidarta‐Oliveira , A. Caron , S. A. Stanley , A. I. Domingos , Neuron 2022, 110, 3597. 36327900 10.1016/j.neuron.2022.10.017 PMC9986959

[3] T. O. Mundinger , Q. Mei , A. K. Foulis , C. L. Fligner , R. L. Hull , G. J. Taborsky Jr. , Diabetes 2016, 65, 2322. 27207540 10.2337/db16-0284 PMC4955989

[4] S. Li , H. Yuan , K. Yang , Q. Li , M. Xiang , Clin. Immunol. 2023, 250, 109319. 37024024 10.1016/j.clim.2023.109319

[5] G. Christoffersson , S. S. Ratliff , Sci. Adv. 2020, 6, abb2878. 10.1126/sciadv.abb2878 PMC7531904 33052874

[6] Z. Msheik , M. El Massry , A. Rovini , F. Billet , A. Desmouliere , J. Neuroinflamm. 2022, 19, 97. 10.1186/s12974-022-02454-6 PMC9013246 35429971

[7] B. Zhao , Y. Zhao , X. Sun , Pharmacol. Res. 2024, 210, 107505. 39547465 10.1016/j.phrs.2024.107505

[8] D. Strasser , K. Neumann , H. Bergmann , M. J. Marakalala , R. Guler , A. Rojowska , K. P. Hopfner , F. Brombacher , H. Urlaub , G. Baier , G. D. Brown , M. Leitges , J. Ruland , Immunity 2012, 36, 32. 22265677 10.1016/j.immuni.2011.11.015 PMC3477316

[9] H. Hara , T. Saito , Trends Immunol. 2009, 30, 234. 19359218 10.1016/j.it.2009.03.002

[10] Z. Xu , D. Li , W. Qu , Y. Yin , S. Qiao , Y. Zhu , S. Shen , Y. Hou , J. Yang , T. Wang , Cell Death Dis. 2022, 13, 502. 35618701 10.1038/s41419-022-04938-y PMC9135688

[11] M. Yang , J. H. Shao , Y. J. Miao , W. Cui , Y. F. Qi , J. H. Han , X. Lin , J. Du , Cell Death Differ. 2014, 21, 1290. 24722209 10.1038/cdd.2014.45 PMC4085533

[12] X. Liu , B. Jiang , H. Hao , Z. Liu , Front. Immunol. 2022, 13, 880879. 35432375 10.3389/fimmu.2022.880879 PMC9005907

[13] H. Ennerfelt , C. Holliday , D. A. Shapiro , K. E. Zengeler , A. C. Bolte , T. K. Ulland , J. R. Lukens , Proc. Natl. Acad. Sci. U S A 2023, 120, 2303760120. 10.1073/pnas.2303760120 PMC10268238 37276426

[14] L. Kazak , P. Cohen , Nat. Rev. Endocrinol. 2020, 16, 421. 32493980 10.1038/s41574-020-0365-5

[15] L. Ji , X. Zhao , B. Zhang , L. Kang , W. Song , B. Zhao , W. Xie , L. Chen , X. Hu , Immunity 2019, 51, 272. 31399282 10.1016/j.immuni.2019.06.007

[16] S. C. Forbes , D. M. Cordingley , S. M. Cornish , B. Gualano , H. Roschel , S. M. Ostojic , E. S. Rawson , B. D. Roy , K. Prokopidis , P. Giannos , D. G. Candow , Nutrients 2022, 14, 921. 35267907 10.3390/nu14050921 PMC8912287

[17] J. Gao , T. Dharmadasa , A. Malaspina , P. J. Shaw , K. Talbot , M. R. Turner , A. G. Thompson , J. Neurol. 2022, 269, 5395. 35614165 10.1007/s00415-022-11195-8 PMC9467954

[18] M. Kvist‐Reimer , F. Sundler , B. Ahren , Cell Tissue Res. 2002, 307, 203. 11845327 10.1007/s00441-001-0496-5

[19] F. D. Allman , E. L. Rogers , D. A. Caniano , D. M. Jacobowitz , M. C. Rogers , Crit. Care Med. 1982, 10, 100. 6800697 10.1097/00003246-198202000-00006

[20] J. Meng , H. Chen , Q. Lv , X. Luo , K. Yang , Med. Sci. Monit. 2020, 26, 922986. 10.12659/MSM.922986 PMC7433386 32764532

[21] M. Li , J. Galligan , D. Wang , G. Fink , Auton. Neurosci. 2010, 154, 66. 20053590 10.1016/j.autneu.2009.11.009

[22] L. M. Roberts , H. E. Ledvina , S. Tuladhar , D. Rana , S. P. Steele , G. D. Sempowski , J. A. Frelinger , Immun. Inflamm. Dis. 2015, 3, 71. 26029367 10.1002/iid3.51 PMC4444150

[23] J. Lee , D. H. Hyun , Antioxidants 2023, 12, 918.37107292

[24] Z. Zou , R. Liu , Y. Wang , H. Tan , G. An , B. Zhang , Y. Wang , D. Dong , CNS Neurosci. Ther. 2023, 29, 2145. 36914965 10.1111/cns.14162 PMC10352898

[25] S. An , J. Shi , J. Huang , Z. Li , M. Feng , G. Cao , Mol. Neurobiol. 2024, 61, 6300. 38291291 10.1007/s12035-024-03964-5

[26] B. R. Stockwell , Cell 2022, 185, 2401. 35803244 10.1016/j.cell.2022.06.003 PMC9273022

[27] T. W. Lu , J. Wu , P. C. Aoto , J. H. Weng , L. G. Ahuja , N. Sun , C. Y. Cheng , P. Zhang , S. S. Taylor , Proc. Natl. Acad. Sci. U S A 2019, 116, 16347. 31363049 10.1073/pnas.1906036116 PMC6697891

[28] T. C. Fung , C. A. Olson , E. Y. Hsiao , Nat. Neurosci. 2017, 20, 145. 28092661 10.1038/nn.4476 PMC6960010

[29] J. Wang , J. Gu , F. Ma , Y. Wei , P. Wang , S. Yang , X. Yan , Y. Xiao , K. Xing , A. Lou , L. Zheng , T. Cao , D. Zhu , J. Li , L. Zhang , Y. Li , T. Chen , Research 2024, 7, 0493. 39381792 10.34133/research.0493 PMC11458856

[30] W. Ren , M. Hua , F. Cao , W. Zeng , Adv. Sci. 2024, 11, 2306128. 10.1002/advs.202306128 PMC10885671 38039489

[31] E. L. Feldman , B. C. Callaghan , R. Pop‐Busui , D. W. Zochodne , D. E. Wright , D. L. Bennett , V. Bril , J. W. Russell , V. Viswanathan , Nat. Rev. Dis. Primers 2019, 5, 41. 31197153 10.1038/s41572-019-0092-1

[32] Y. Yu , H. Chen , S. B. Su , J. Neuroinflamm. 2015, 12, 141. 10.1186/s12974-015-0368-7 PMC4527131 26245868

[33] T. O. Mundinger , G. J. Taborsky Jr. , Diabetologia 2016, 59, 2058. 27342407 10.1007/s00125-016-4026-0 PMC6214182

[34] Z. Zhang , L. He , S. Hu , Y. Wang , Q. Lai , P. Yang , Q. Yu , S. Zhang , F. Xiong , S. Simsekyilmaz , Q. Ning , J. Li , D. Zhang , H. Zhang , X. Xiang , Z. Zhou , H. Sun , C. Y. Wang , Am. J. Transl. Res. 2015, 7, 1812. 26692926 PMC4656759

[35] M. Pereira , P. Tourlomousis , J. Wright , T. P. Monie , C. E. Bryant , Nat. Commun. 2016, 7, 12874. 27670879 10.1038/ncomms12874 PMC5052644

[36] C. Tian , H. Yuan , Y. Lu , H. He , Q. Li , S. Li , J. Yang , M. Wang , R. Xu , Q. Liu , M. Xiang , Oncoimmunology 2023, 12, 2204015. 37089447 10.1080/2162402X.2023.2204015 PMC10120541

[37] G. J. Taborsky , Q. Mei , D. J. Hackney , D. P. Figlewicz , R. LeBoeuf , T. O. Mundinger , Diabetologia 2009, 52, 2602. 19798480 10.1007/s00125-009-1494-5

[38] G. J. Taborsky , Q. Mei , K. E. Bornfeldt , D. J. Hackney , T. O. Mundinger , Diabetes 2014, 63, 2369. 24608438 10.2337/db13-0778 PMC4066345

[39] G. Pongratz , R. H. Straub , Arthritis Res. Ther. 2014, 16, 504. 25789375 10.1186/s13075-014-0504-2 PMC4396833

[40] I. Gabanyi , P. A. Muller , L. Feighery , T. Y. Oliveira , F. A. Costa‐Pinto , D. Mucida , Cell 2016, 164, 378. 26777404 10.1016/j.cell.2015.12.023 PMC4733406

[41] K. Sakamoto , M. A. Butera , C. Zhou , G. Maurizi , B. Chen , L. Ling , A. Shawkat , L. Patlolla , K. Thakker , V. Calle , D. A. Morgan , K. Rahmouni , G. J. Schwartz , A. Tahiri , C. Buettner , Cell Metab. 2025, 37, 121. 39437790 10.1016/j.cmet.2024.09.012 PMC11711004

[42] A. Alvarsson , M. Jimenez‐Gonzalez , R. Li , C. Rosselot , N. Tzavaras , Z. Wu , S. A. Stanley , Bio Protocol 2021, 11, 4103. 10.21769/BioProtoc.4103 PMC8376577 34458397

[43] D. Villarreal , G. Pradhan , C. S. Wu , C. D. Allred , S. Guo , Y. Sun , J. Vis. Exp. 2019, 150, 57048. 10.3791/57048 PMC7954665 31524856

[44] M. I. Love , W. Huber , S. Anders , Genome Biol. 2014, 15, 550. 25516281 10.1186/s13059-014-0550-8 PMC4302049

[45] T. Wu , E. Hu , S. Xu , M. Chen , P. Guo , Z. Dai , T. Feng , L. Zhou , W. Tang , L. Zhan , X. Fu , S. Liu , X. Bo , G. Yu , Innovation 2021, 2, 100141. 34557778 10.1016/j.xinn.2021.100141 PMC8454663

[46] S. Slovin , A. Carissimo , F. Panariello , A. Grimaldi , V. Bouche , G. Gambardella , D. Cacchiarelli , Methods Mol. Biol. 2021, 2284, 343. 33835452 10.1007/978-1-0716-1307-8_19

