# Supplementary material for: CARD9 Conveys Pancreatic Islet Sympathetic Nervous β2 Signals to Reshape Macrophage Creatine Metabolism in Type 1 Diabetes
Source: Adv Sci (Weinh). 2025 Nov 16;13(3):e07543. doi: 10.1002/advs.202507543 (PMC12806524; doi:10.1002/advs.202507543)
Supplement: Supplementary file 1 — Supporting Information [file ADVS-13-e07543-s001.docx]

**CARD9 Conveys Pancreatic Islet Sympathetic Nervous β2 Signals to Reshape Macrophage Creatine Metabolism in Type 1 Diabetes**

**Running title:** CARD9 Modulates T1D via Sympathetic-Macrophage Link​

Huimin Yuan^1, #^, Senlin Li^1, 4, #^, Shuai Liu^2^, Hong Zhou^3^, Yue Xu^1^, Wenhui Li^1^, Mengxuan Wang^1^, Mengheng Wang^1^, Mengzhu Zheng^1^, Wenxi Shi^1^, Xin Li^1^, Yiting Feng^1^, Ming Xiang^1, *^

^1^ Department of Pharmacology, School of Pharmacy, Tongji Medical College, Huazhong University of Science and Technology, Wuhan 430000, China.

^2^ Tongji Hospital, Tongji Medical College, Huazhong University of Science and Technology, Wuhan, 430000, China

^3^ Department of Pharmacy, Union Hospital, Tongji Medical College, Huazhong University of Science and Technology, Wuhan 430022，China

^4^ Institute of Pharmaceutical Innovation, Hubei Province Key Laboratory of Occupational Hazard Identification and Control, School of Medicine, Wuhan University of Science and Technology, Wuhan 430065, China.

**ORCID:**

Ming Xiang: https://orcid.org/0000-0002-3048-3897

Huimin Yuan: https://orcid.org/0009-0006-6253-7585

Senlin Li: https://orcid.org/0000-0002-2665-4171

***Corresponding author:**

Ming Xiang, Department of Pharmacology, School of Pharmacy, Tongji Medical College, Huazhong University of Science and Technology, Hang Kong Road 13, Wuhan 430000, China. Tel/Fax: +86 27 83692784; E-mail: [xiangming@tjmu.edu.cn](mailto:xiangming@tjmu.edu.cn).

^#^These authors contributed equally to this work.

**Supplementary Materials**

This PDF file includes:

Figs. S1 to S16

Tables S1 to S4


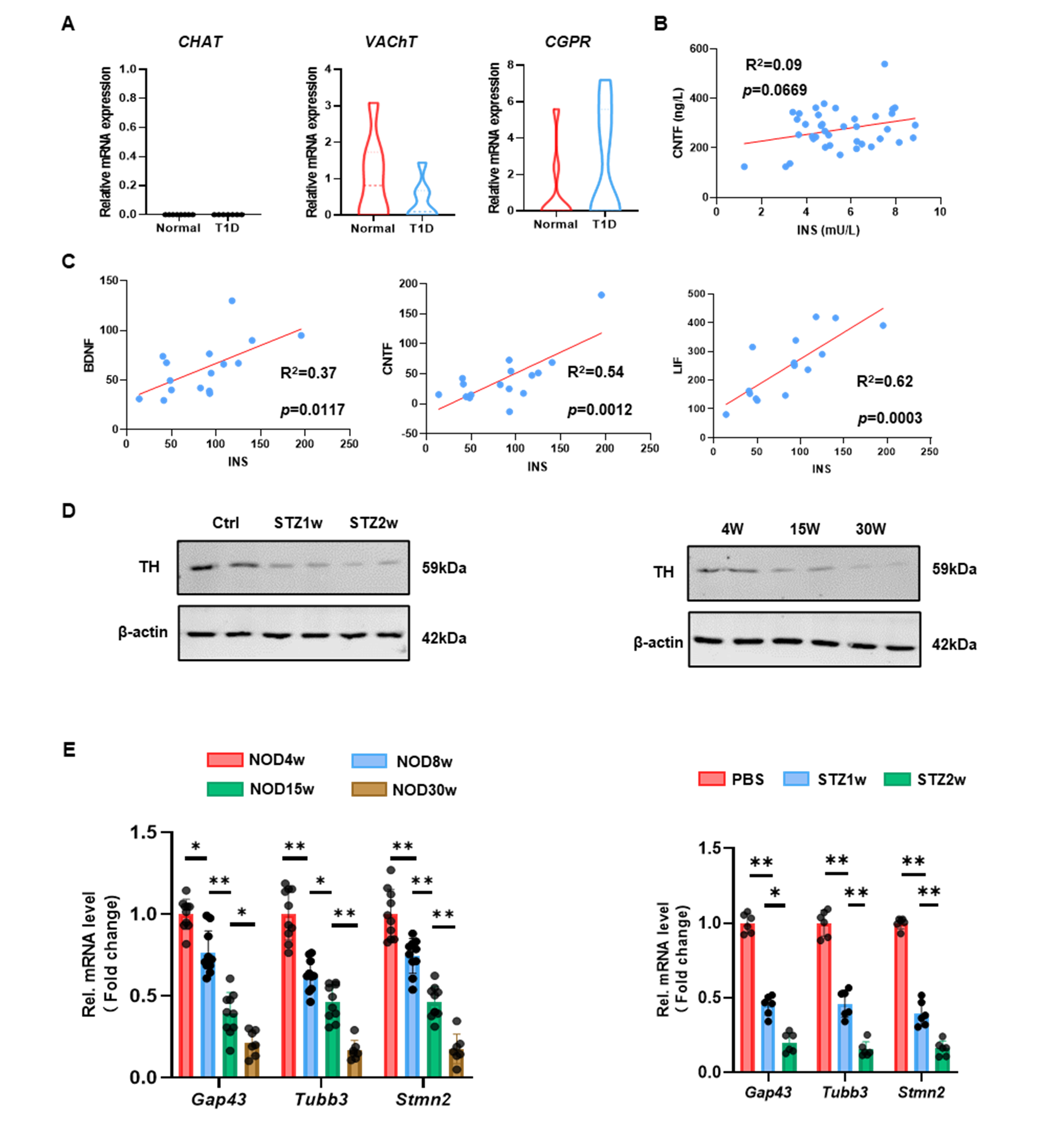
**Fig. S1 Alterations of neurotrophic and neuronal markers in T1D.** (A) Bar chart illustrating the expression levels of *CHAT*, *VAChT*, and *CGRP* in pancreatic islet tissues of normal individuals and T1D patients. (B) A scatter plot depicting the correlation between plasma insulin levels and CNTF in diabetic patients. (C) Scatter plot showing the correlations between plasma insulin levels and BDNF, CNTF, as well as LIF in T1D patients from the database. (D) Western blot analysis of TH expression levels in pancreatic tissues of male C57BL/6J mice and normal mice, as well as in pancreatic tissues of female NOD mice at different ages. (E) Expression levels of *Gap43*, *Tubb3*, and *Stmn2* in pancreatic tissues from female NOD mice at different ages, and from male C57BL/6J mice at different time points after STZ-induced T1D. The data were compiled from at least three independent experiments and are presented as the means ± SEMs. **P* < 0.05, ***P* < 0.01, ****P* < 0.001 by Student’s unpaired two-tailed t test or one-way analysis of variance (ANOVA) with Tukey’s post hoc comparison.


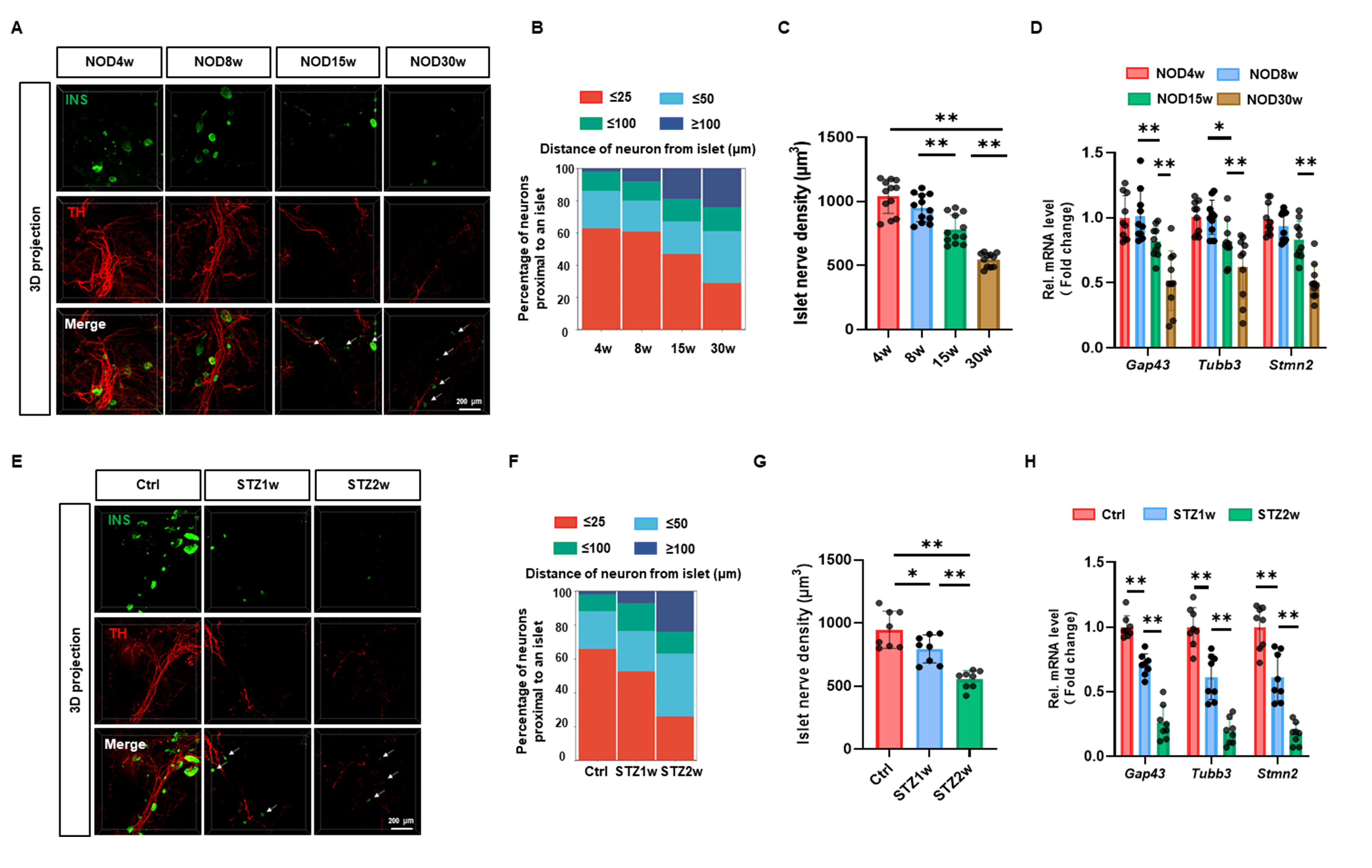


**Fig. S2 Sympathetic nerve alterations in NOD and STZ-induced T1D mouse models.** (A-B) Whole-tissue immunofluorescence of pancreatic tissues from male NOD mice at different ages. Insulin is shown in green, and TH in red. Distances between sympathetic nerve fibers and islets were analyzed and quantified using Imaris software. (C) Quantification of islet sympathetic nerve fiber density in male NOD mice at different ages using Imaris software. (D) Expression levels of *Gap43*, *Tubb3*, and *Stmn2* in pancreatic tissues from male NOD mice at different ages. (E-F) Whole-tissue immunofluorescence of pancreatic tissues from female C57BL/6J mice at different time points after STZ injection to induce T1D. Insulin is shown in green, and TH in red. Distances between sympathetic nerve fibers and islets were analyzed and quantified using Imaris software. (G) Quantification of islet sympathetic nerve fiber density in female C57BL/6J mice at different time points after STZ injection using Imaris software. (H) Expression levels of *Gap43*, *Tubb3*, and *Stmn2* in pancreatic tissues from female C57BL/6J mice at different time points after STZ-induced T1D. The data were compiled from at least three independent experiments and are presented as the means ± SEMs. **P* < 0.05, ***P* < 0.01, ****P* < 0.001 by Student’s unpaired two-tailed t test or one-way analysis of variance (ANOVA) with Tukey’s post hoc comparison.


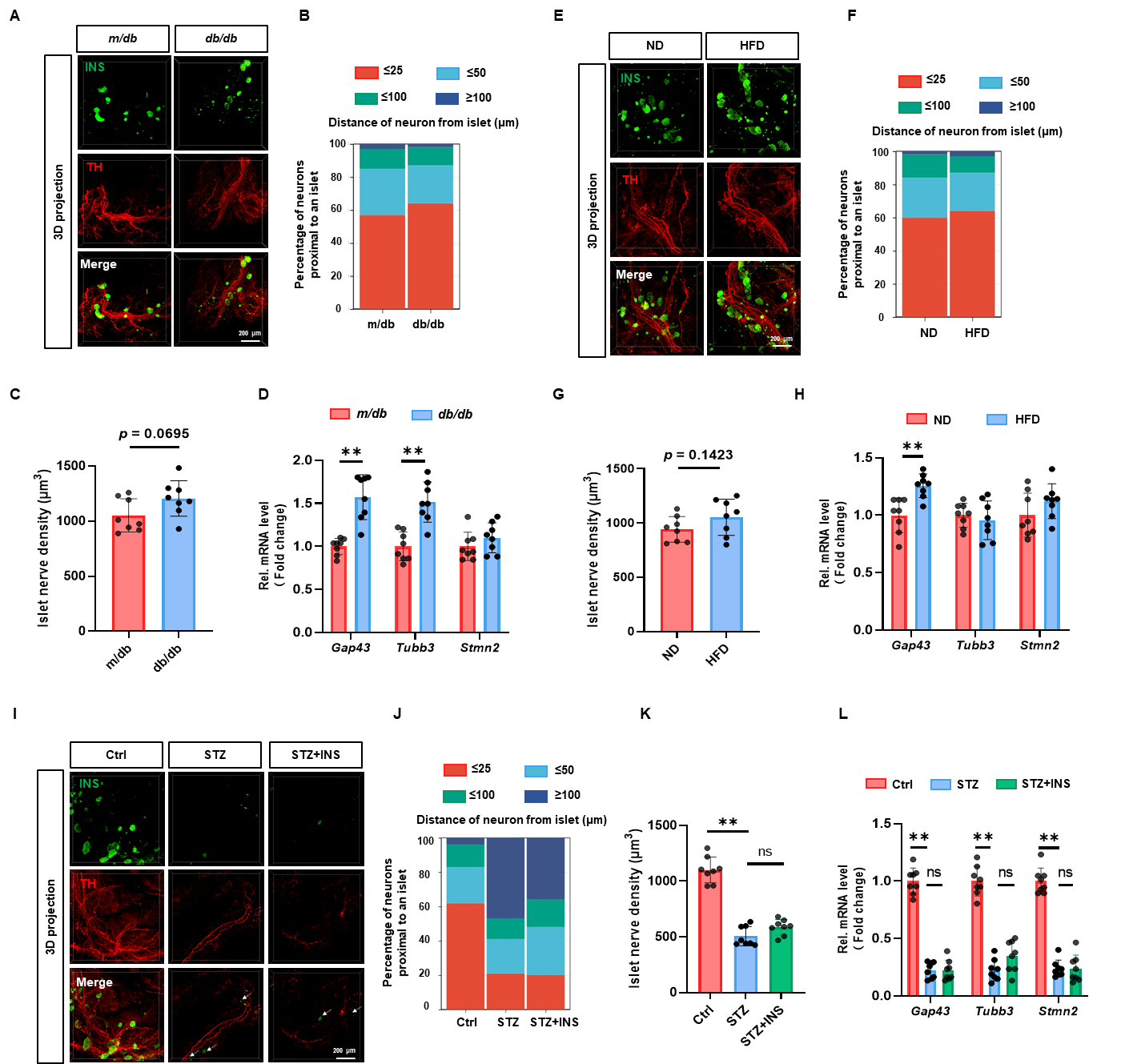


**Figure S3. Sympathetic nerve alterations in diabetes models.** (A-B) Whole-tissue immunofluorescence of pancreatic tissues from *db/db* mice. Insulin is shown in green, and TH in red. Distances between sympathetic nerve fibers and islets were analyzed and quantified using Imaris software. (C) Quantification of islet sympathetic nerve fiber density in *db/db* mice using Imaris software. (D) Expression levels of *Gap43*, *Tubb3*, and *Stmn2* in pancreatic tissues from *db/db* mice. (E-F) Whole-tissue immunofluorescence of pancreatic tissues from high-fat diet–induced T2D mice. Insulin is shown in green, and TH in red. Distances between sympathetic nerve fibers and islets were analyzed and quantified using Imaris software. (G) Quantification of islet sympathetic nerve fiber density in high-fat diet–induced T2D mice using Imaris software. (H) Expression levels of *Gap43*, *Tubb3*, and *Stmn2* in pancreatic tissues from high-fat diet–induced T2D mice. (I-J) Whole-tissue immunofluorescence of pancreatic tissues from STZ-induced T1D mice receiving long-acting insulin (insulin glargine) to maintain stable blood glucose and insulin levels. Insulin is shown in green, and TH in red. Distances between sympathetic nerve fibers and islets were analyzed and quantified using Imaris software. (K) Quantification of islet sympathetic nerve fiber density in STZ-induced T1D mice after insulin treatment using Imaris software. (L) Expression levels of *Gap43*, *Tubb3*, and *Stmn2* in pancreatic tissues from STZ-induced T1D mice after insulin treatment. The data were compiled from at least three independent experiments and are presented as the means ± SEMs. **P* < 0.05, ***P* < 0.01, ****P* < 0.001 by Student’s unpaired two-tailed t test or one-way analysis of variance (ANOVA) with Tukey’s post hoc comparison.


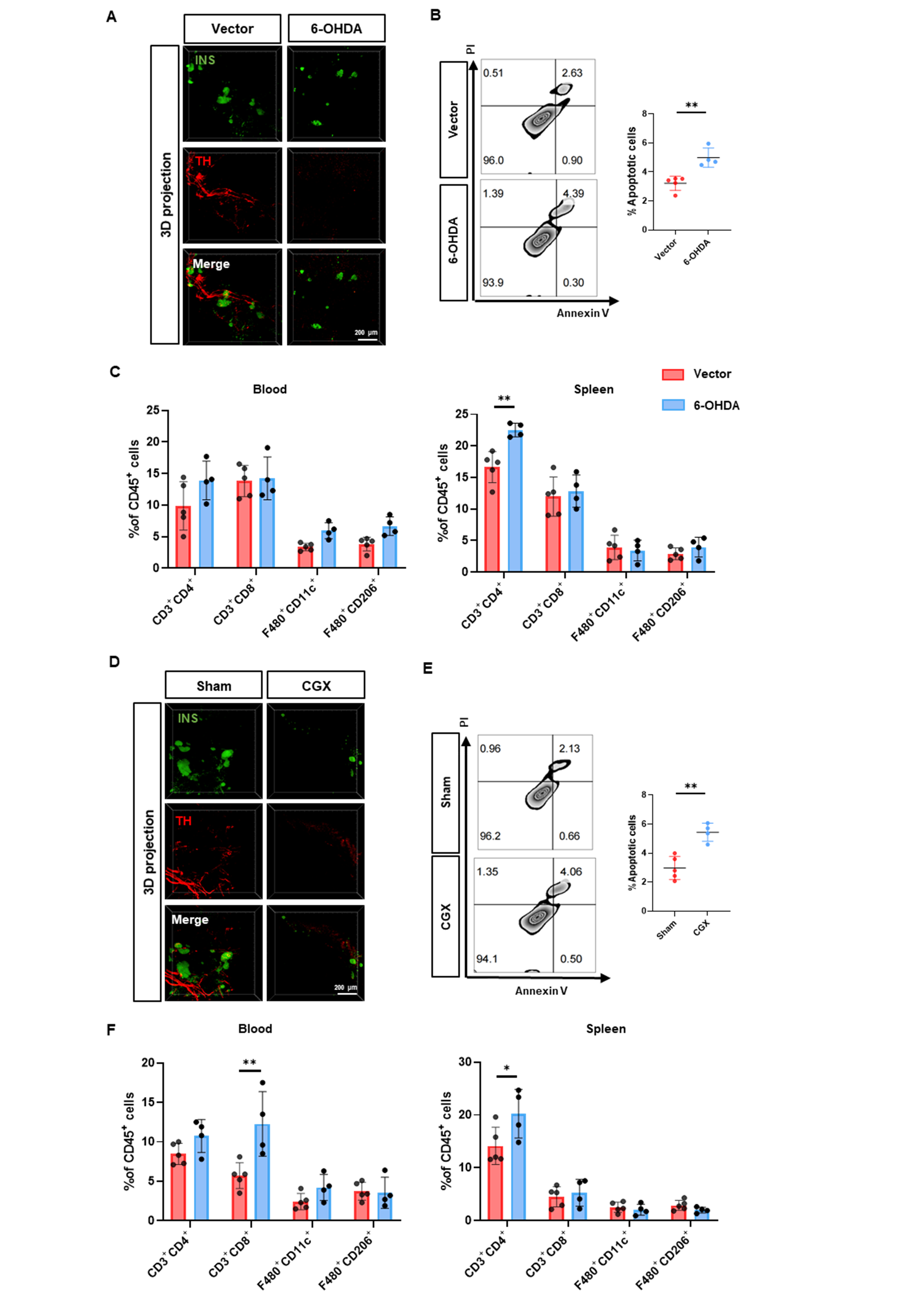


**Fig. S4 Effects of sympathetic denervation on pancreatic apoptosis and immune cell populations in NOD mice.** (A) Whole-tissue immunofluorescence imaging of pancreatic tissue in NOD mice injected with 6-OHDA, with insulin shown in green and TH in red. (B) Apoptosis analysis of pancreatic single cells. (C) Flow cytometry analysis of immune cell populations in the spleen, and peripheral blood, quantifying the proportions of CD3^+^CD4^+^, CD3^+^CD8^+^, F4/80^+^CD11c^+^, and F4/80^+^CD206^+^ cells. (D) Whole-tissue immunofluorescence imaging of pancreatic tissue in NOD mice underwent CGX surgery and sham surgery, with insulin shown in green and TH in red. (E) Apoptosis analysis of pancreatic single cells. (F) Flow cytometry analysis of immune cell populations in the spleen, and peripheral blood, quantifying the proportions of CD3^+^CD4^+^, CD3^+^CD8^+^, F4/80^+^CD11c^+^, and F4/80^+^CD206^+^ cells. The data were compiled from at least three independent experiments and are presented as the means ± SEMs. **P* < 0.05, ***P* < 0.01, ****P* < 0.001 by Student’s unpaired two-tailed t test or one-way analysis of variance (ANOVA) with Tukey’s post hoc comparison.


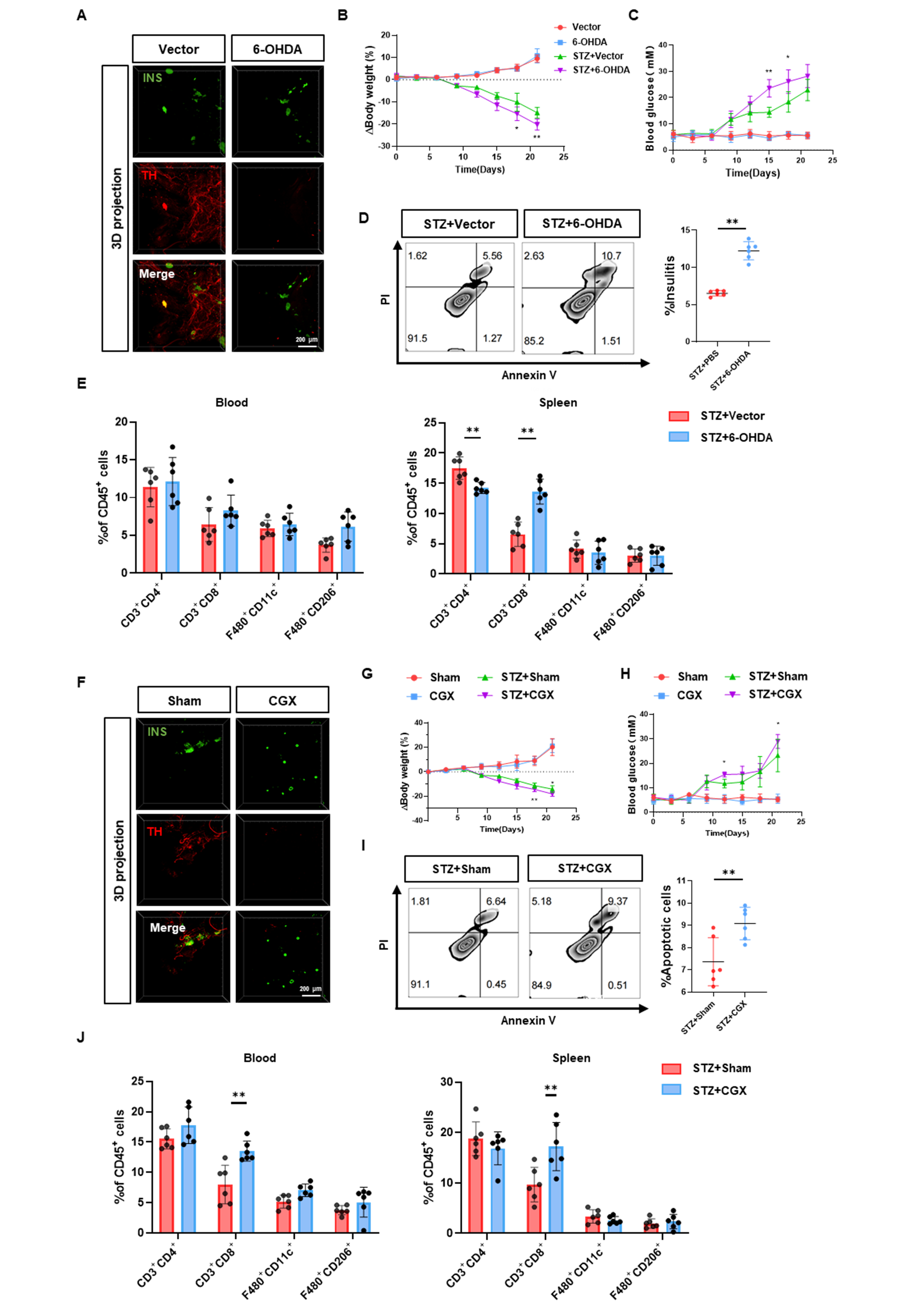


**Fig. S5 Effects of sympathetic denervation on metabolic and immune parameters in STZ induced T1D C57BL/6J model mice.** (A) Whole-tissue immunofluorescence imaging of pancreatic tissue in C57BL/6J mice injected with 6-OHDA, with insulin shown in green and TH in red. (B) Weight changes. (C) Changes in blood glucose levels. (D) Apoptosis analysis of pancreatic single cells. (E) Flow cytometry analysis of immune cell populations in the spleen, and peripheral blood, quantifying the proportions of CD3^+^CD4^+^, CD3^+^CD8^+^, F4/80^+^CD11c^+^, and F4/80^+^CD206^+^ cells. (F) Whole-tissue immunofluorescence imaging of pancreatic tissue in C57BL/6J mice after CGX surgery, with insulin shown in green and TH in red. (G) Weight changes. (H) Changes in blood glucose levels. (I) Apoptosis analysis of pancreatic single cells. (J) Flow cytometry analysis of immune cell populations in the spleen, and peripheral blood, quantifying the proportions of CD3^+^CD4^+^, CD3^+^CD8^+^, F4/80^+^CD11c^+^, and F4/80^+^CD206^+^ cells. The data were compiled from at least three independent experiments and are presented as the means ± SEMs. **P* < 0.05, ***P* < 0.01, ****P* < 0.001 by Student’s unpaired two-tailed t test or one-way analysis of variance (ANOVA) with Tukey’s post hoc comparison.


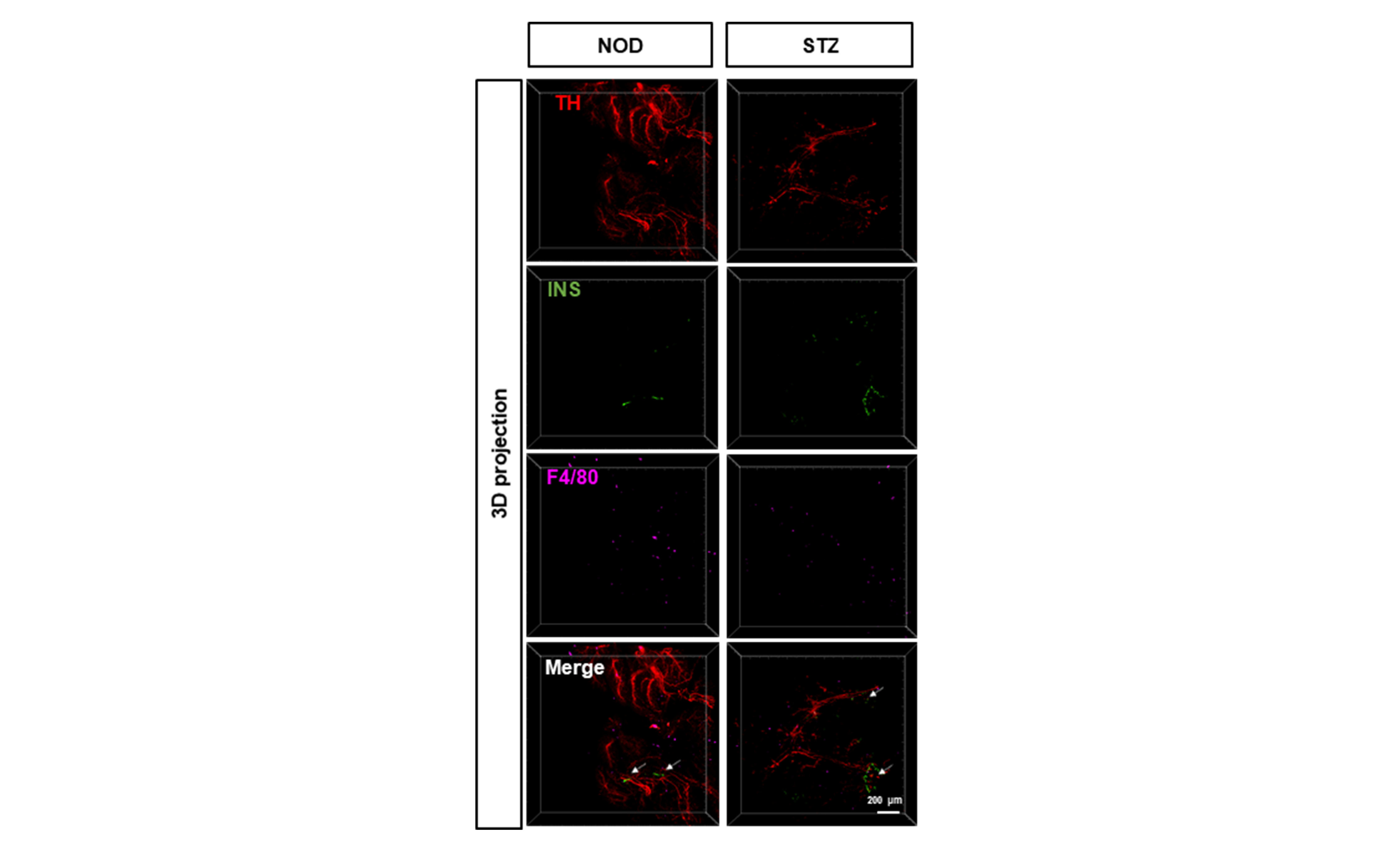


**Fig. S6 Whole-tissue immunofluorescence of pancreatic tissues from T1D and STZ-induced NOD mice.** Insulin is shown in green, TH in red, and F4/80 in purple.


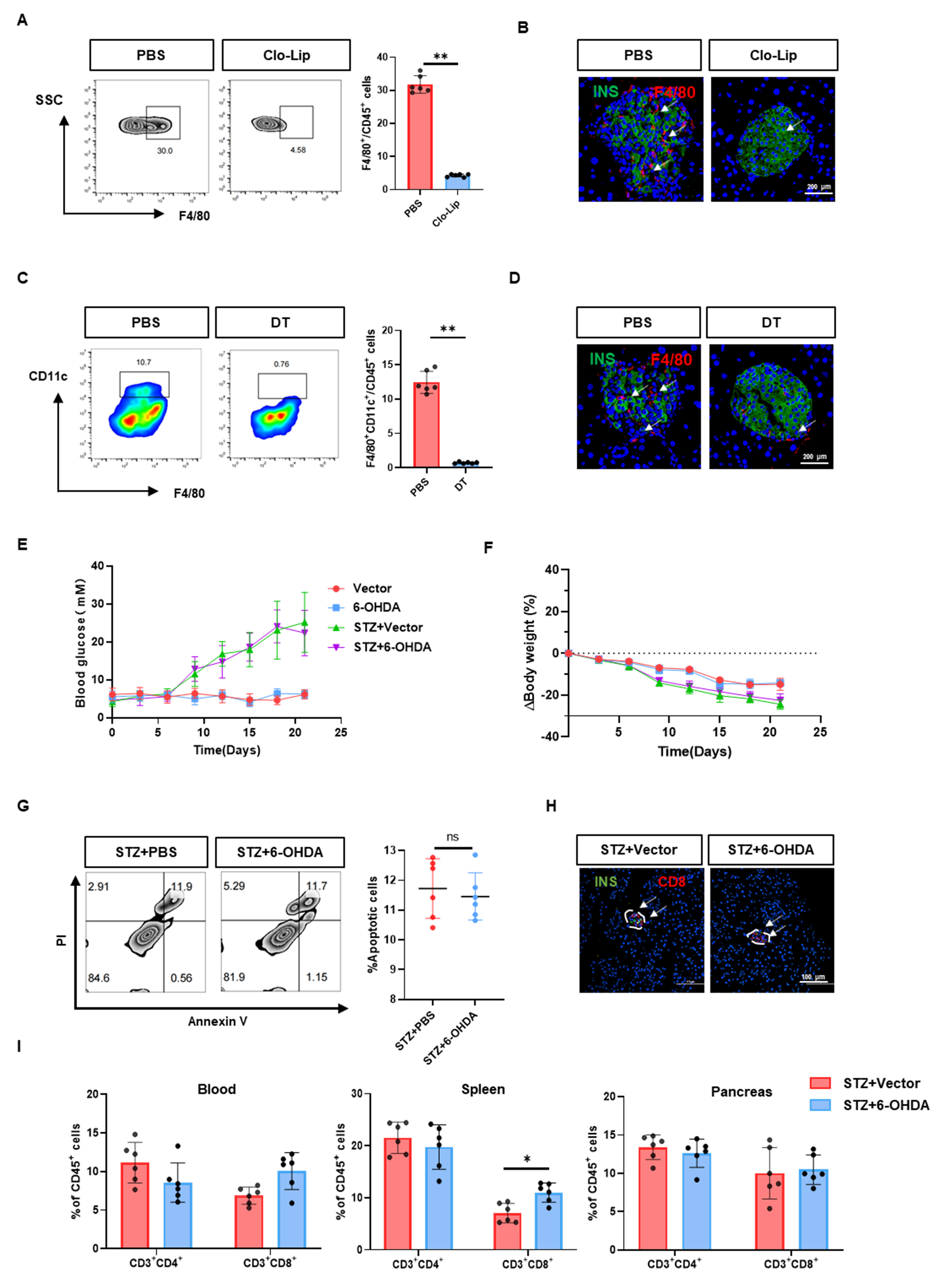


**Fig. S7 Effects of macrophage depletion on pancreatic immune cell populations and metabolic parameters in T1D.** (A) Flow cytometry analysis of pancreatic macrophage proportions in NOD mice after intraductal injection of clodronate liposomes. (B) Immunofluorescence analysis of pancreatic islets in NOD mice after intraductal injection of clodronate liposomes, showing macrophage distribution. (C) Flow cytometry analysis of pancreatic macrophage proportions in CD11c-DTR mice following tail vein injection of diphtheria toxin (DT). (D) Immunofluorescence analysis of pancreatic islets in CD11c-DTR mice after DT injection. (E-F) Blood glucose levels (E) and body weight changes (F) in different mouse groups. (G) Apoptosis analysis of pancreatic single cells. (H) Representative immunofluorescence images showing insulin and CD8 expression in pancreatic tissues. (I) Flow cytometry analysis of immune cell populations in the spleen, peripheral blood, and pancreatic tissues, showing the proportions of CD3^+^CD4^+^, CD3^+^CD8^+^, F4/80^+^CD11c^+^, and F4/80^+^CD206^+^ cells. The data were compiled from at least three independent experiments and are presented as the means ± SEMs. **P* < 0.05, ***P* < 0.01, ****P* < 0.001 by Student’s unpaired two-tailed t test or one-way analysis of variance (ANOVA) with Tukey’s post hoc comparison.


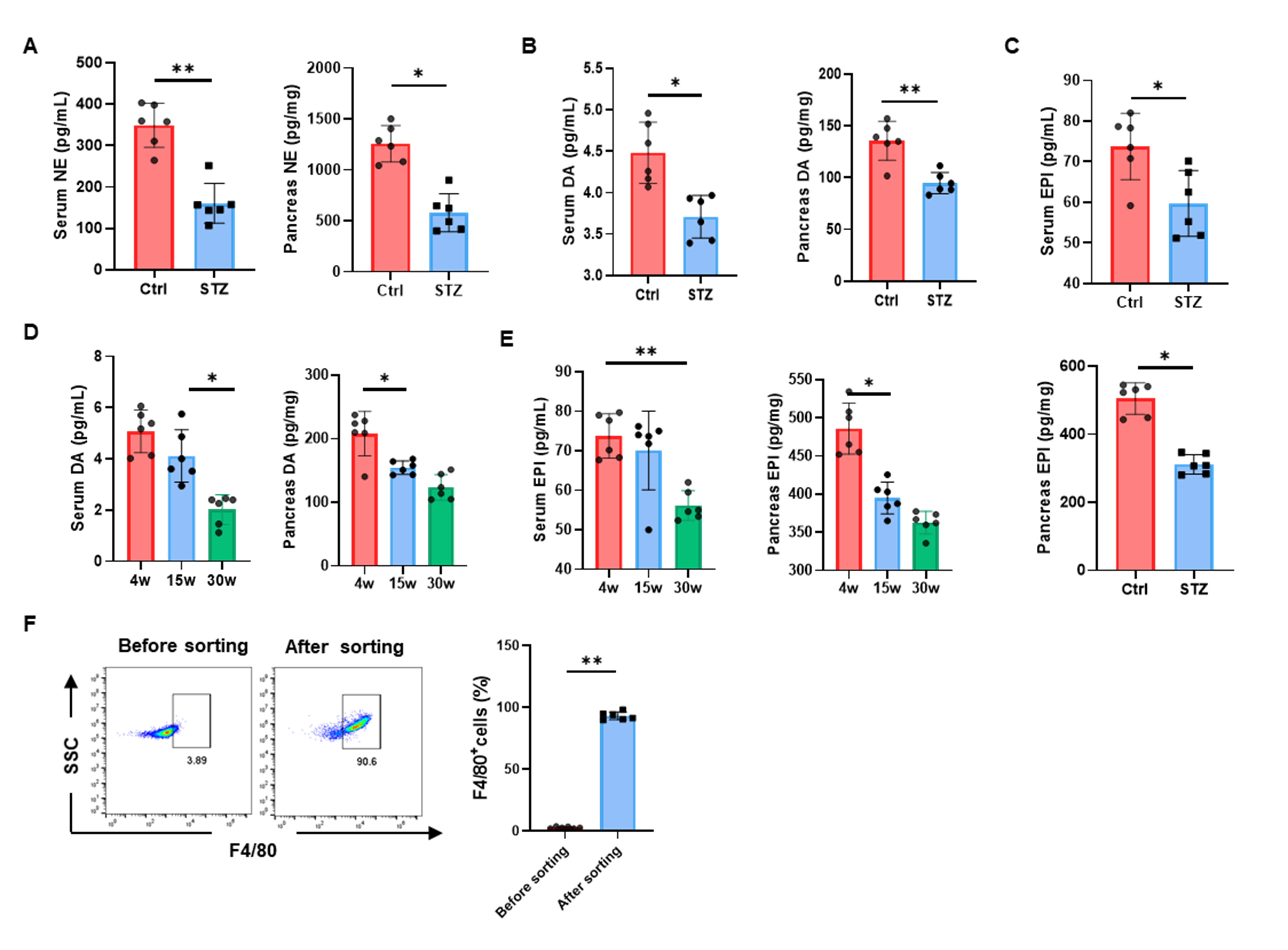


**Fig. S8 Catecholamine levels in diabetic and NOD mice.** (A) NE, (B) DA and (C) EPI levels in the serum and pancreatic tissues of STZ-induced diabetic mice compared to control mice. (D) DA and (E) EPI levels in the serum and pancreatic tissue of NOD mice at different ages. (F) Purity of F4/80^+^ macrophages isolated from pancreatic islets using magnetic bead-based selection, as assessed by flow cytometry. The data were compiled from at least three independent experiments and are presented as the means ± SEMs. **P* < 0.05, ***P* < 0.01, ****P* < 0.001 by Student’s unpaired two-tailed t test or one-way analysis of variance (ANOVA) with Tukey’s post hoc comparison.


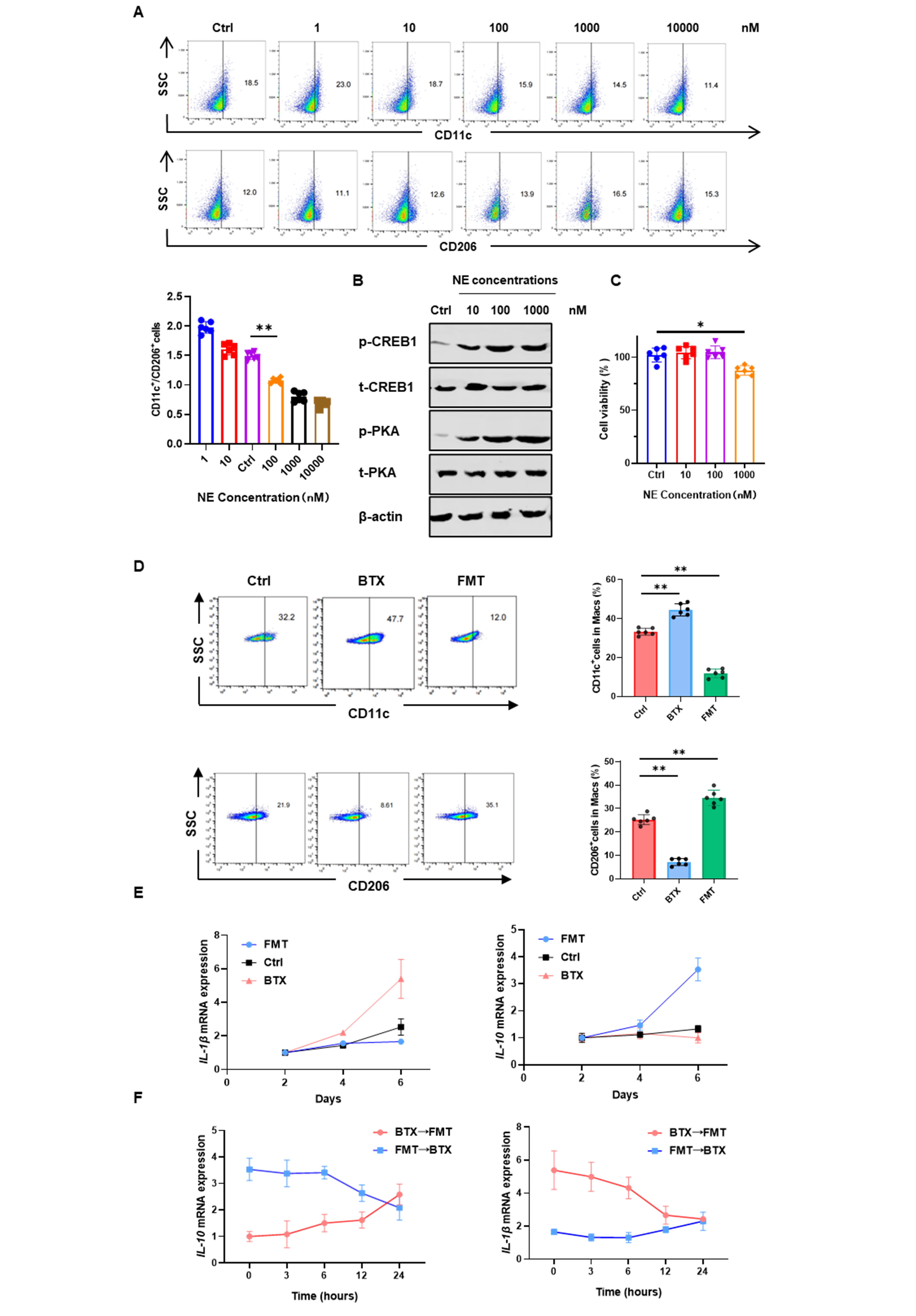


**Figure S9. Effects of NE, FMT, and BTX on BMDMs.** (A) Flow cytometry analysis of BMDMs treated with different concentrations of NE, using F4/80, CD11c, and CD206 as markers. (B) Western blot analysis of PKA, p-PKA, CREB1, and p-CREB1 expression in BMDMs treated with 10, 100, or 1000 nM NE. (C) Cell viability assay of BMDMs treated with 10, 100, or 1000 nM NE. (D) Flow cytometry analysis of BMDM phenotypes after treatment with FMT and BTX, using F4/80, CD11c, and CD206 as markers. (E) qPCR analysis of IL-1β and IL-10 expression in BMDMs at different time points following treatment with FMT and BTX. (F) qPCR analysis of IL-1β and IL-10 expression in BMDMs at different time points after medium replacement following NE treatment. The data were compiled from at least three independent experiments and are presented as the means ± SEMs. **P* < 0.05, ***P* < 0.01, ****P* < 0.001 by Student’s unpaired two-tailed t test or one-way analysis of variance (ANOVA) with Tukey’s post hoc comparison.


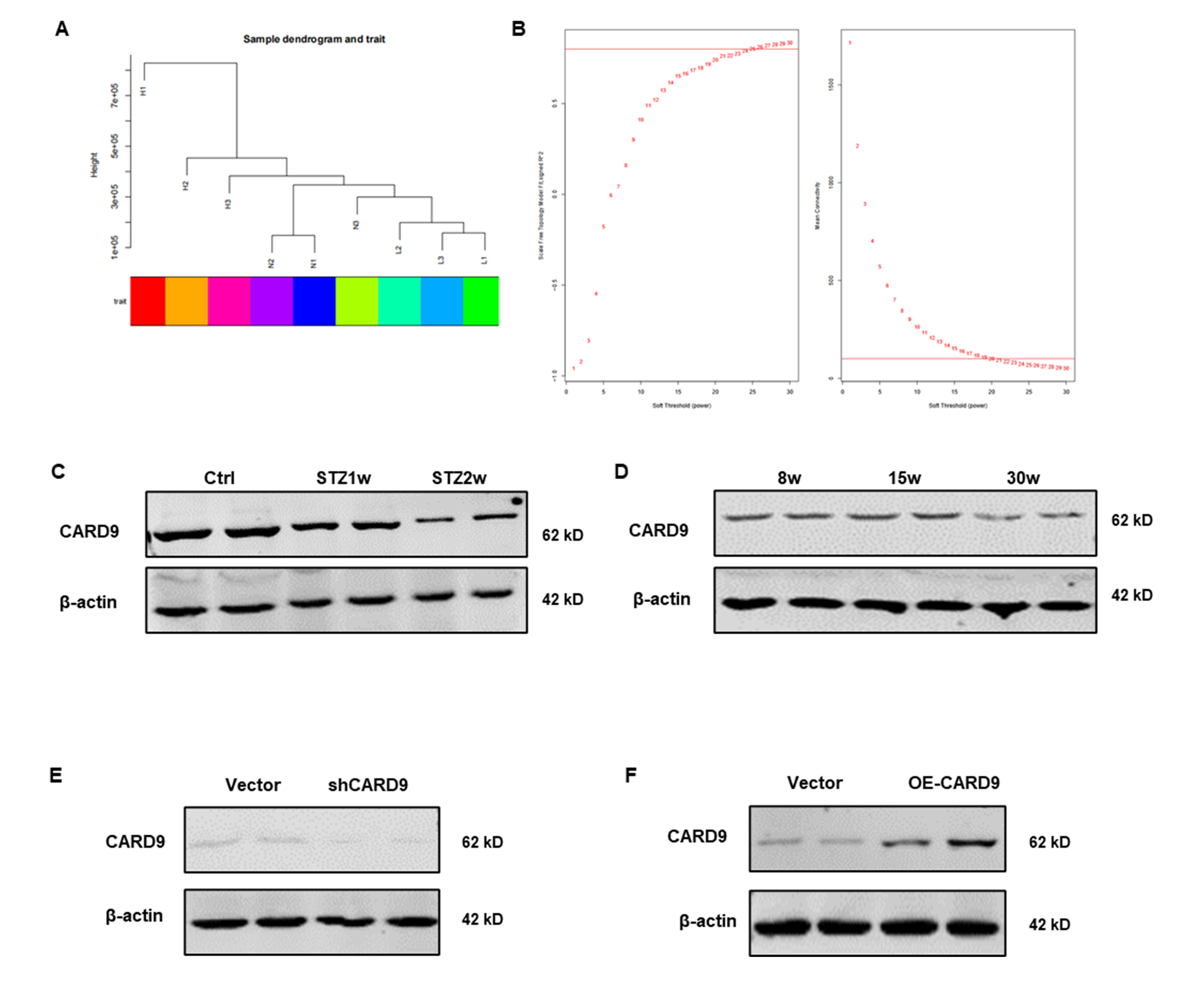


**Fig. S10 CARD9 expression and WGCNA analysis.**

(A) Sample clustering tree in WGCNA analysis. (B) Scale-free network assessment in WGCNA analysis. (C) Western blot analysis of CARD9 protein expression in pancreatic tissues macrophage from STZ-induced T1D mice compared to normal controls. (D) Western blot analysis of CARD9 expression in pancreatic tissues macrophage of NOD mice at different ages. (E-F) Protein level validation of knocking down and overexpressing CARD9.


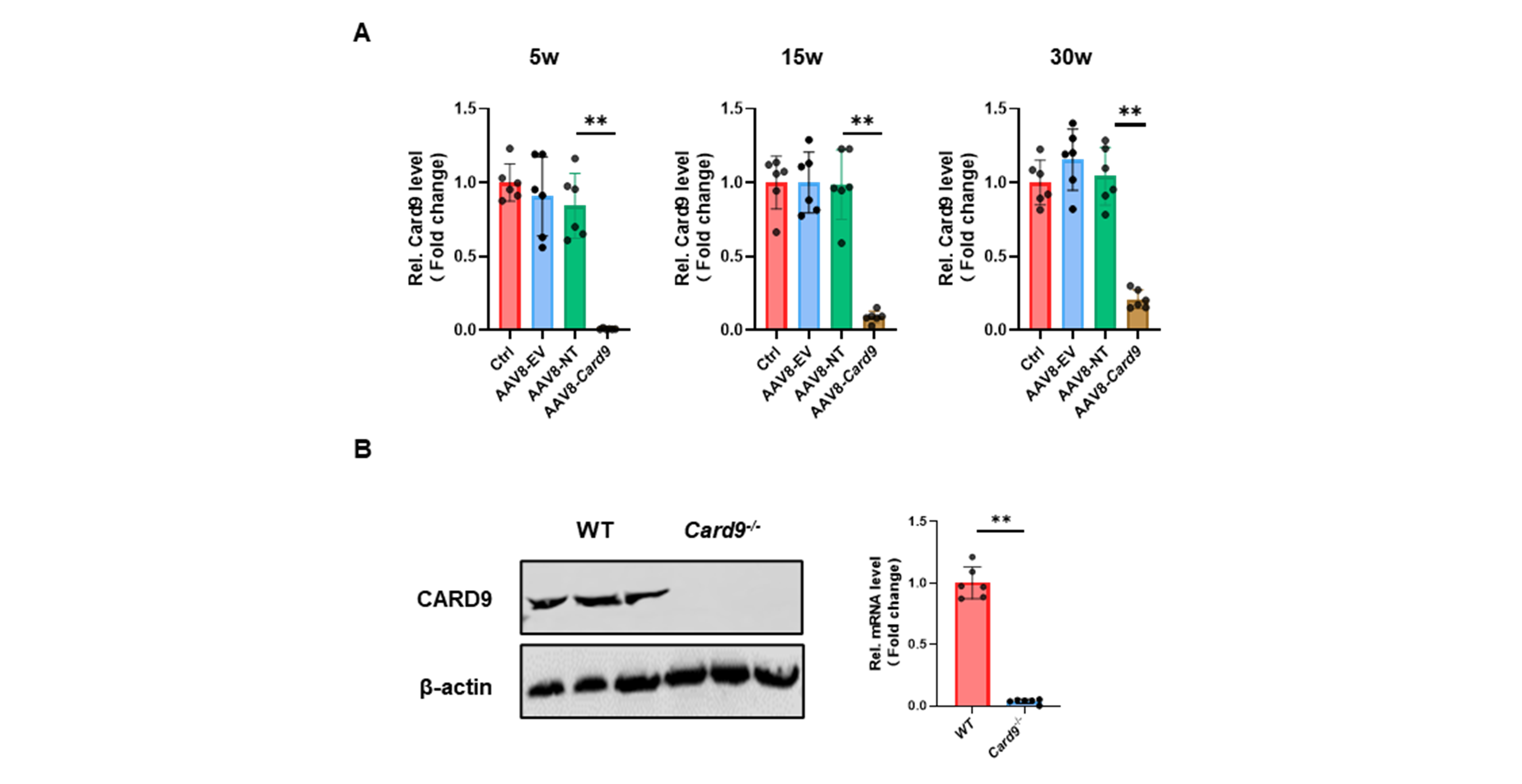


**Figure S11. Validation of CARD9 knockdown and knockout efficiency.** (A) Verification of CARD9 knockdown efficiency in islet macrophages isolated from NOD mice injected with AAV8-sgCard9 at 5, 15, and 30 weeks of age. (B) Gene and protein expression of CARD9 in BMDMs from WT and Card9^−/−^ mice. The data were compiled from at least three independent experiments and are presented as the means ± SEMs. **P* < 0.05, ***P* < 0.01, ****P* < 0.001 by Student’s unpaired two-tailed t test or one-way analysis of variance (ANOVA) with Tukey’s post hoc comparison.


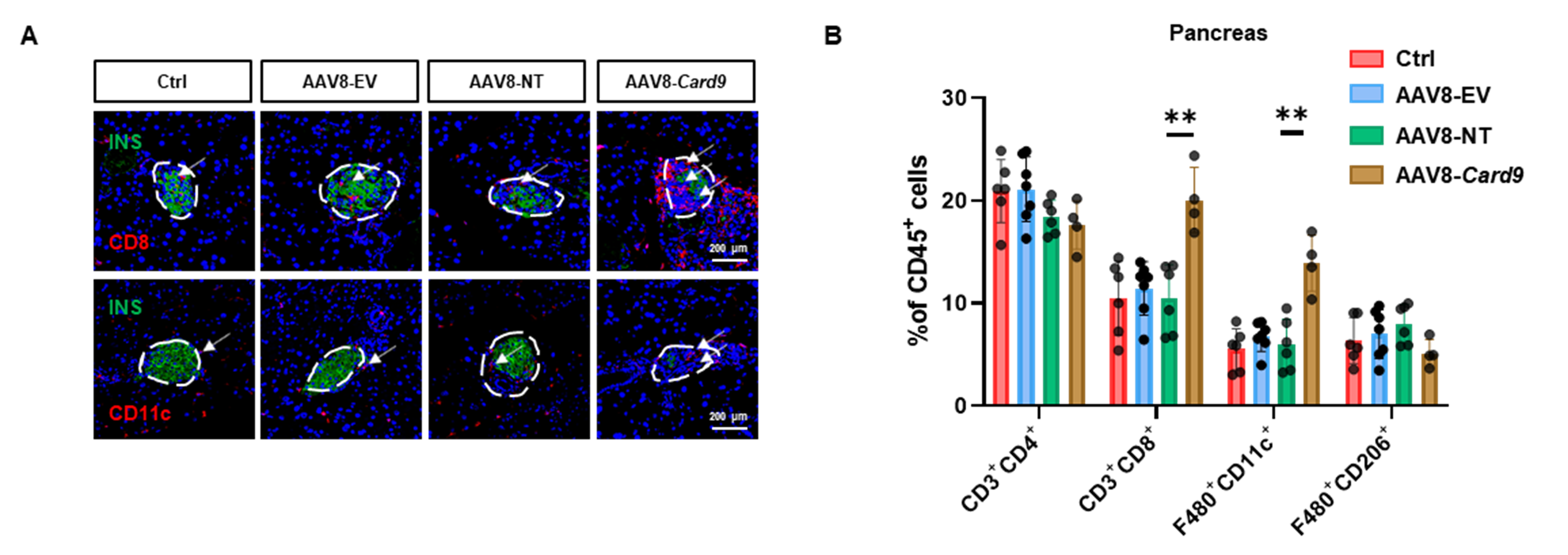


**Fig. S12 Histological and immunological analysis of NOD mice after AAV8-mediated CARD9 depletion in pancreatic macrophages.** (A) Representative immunofluorescence images of pancreatic tissues from NOD mice injected via the pancreatic duct with AAV8-Empty Vector (AAV8-EV), AAV8-Cas9 with non-targeting sgRNA (AAV8-NT), AAV8-Cas9 with sgRNA-*Card9* (AAV8-*Card9*), or saline, showing insulin (INS), CD11c, and CD8 expression. (B) Flow cytometry analysis of immune cell populations in pancreatic tissues, quantifying the proportions of CD3^+^CD4^+^, CD3^+^CD8^+^, F4/80^+^CD11c^+^, and F4/80^+^CD206^+^ cells. The data were compiled from at least three independent experiments and are presented as the means ± SEMs. **P* < 0.05, ***P* < 0.01, ****P* < 0.001 by Student’s unpaired two-tailed t test or one-way analysis of variance (ANOVA) with Tukey’s post hoc comparison.


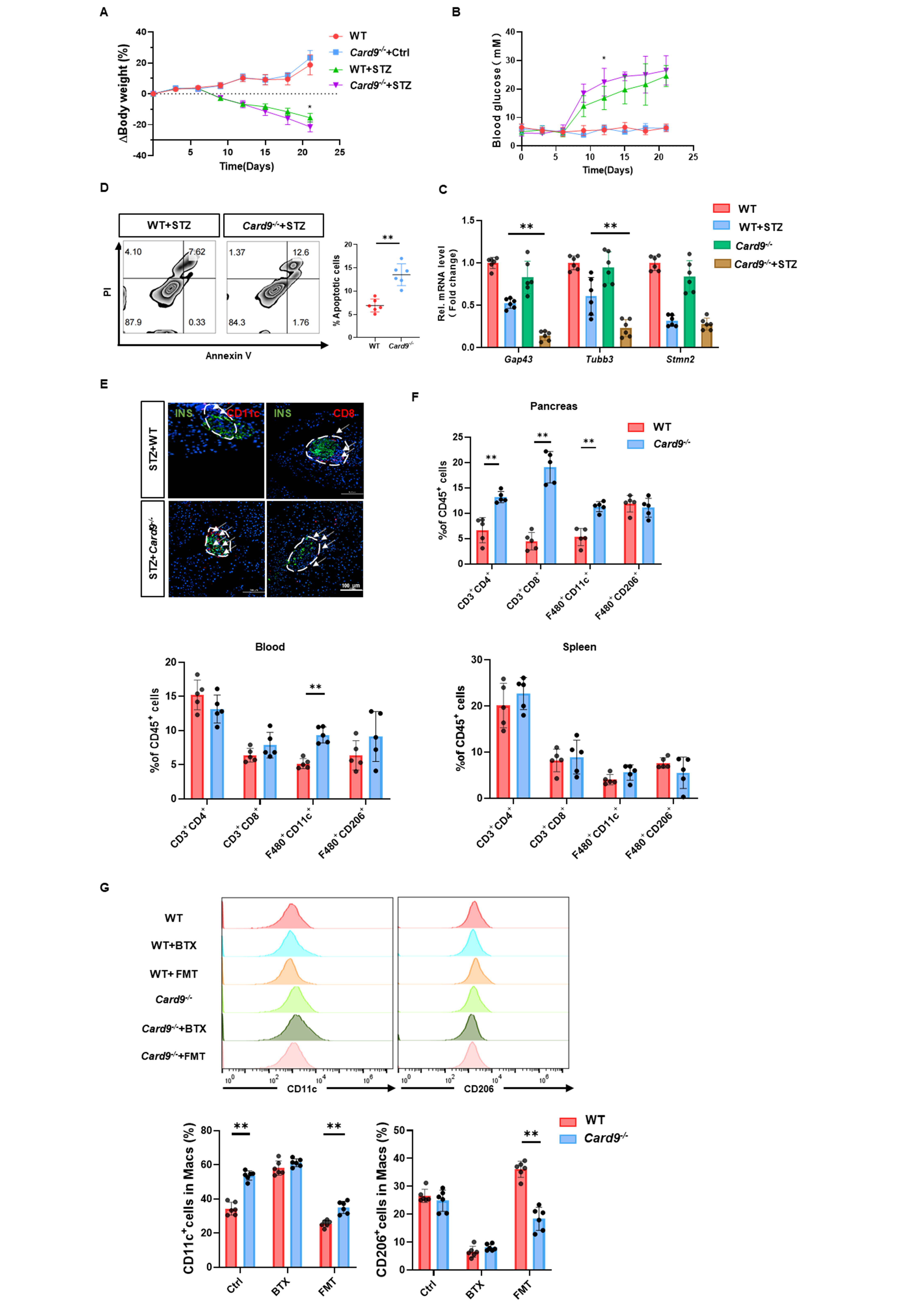


**Fig. S13 Functional and immunological characterization of CARD9-deficient mice and BMDMs.** (A) Body weight changes in WT and Card9^-/-^ mice treated with either citrate buffer or STZ. (B) Blood glucose levels in the indicated groups. (C) Apoptosis analysis of pancreatic single cells. (D) Gene expression levels of *Gap43*, *Tubb3*, and *Stmn2* in pancreatic tissues. (E) Representative immunofluorescence images showing the expression of insulin, CD11c, and CD8 in pancreatic tissues. (F) Flow cytometry analysis of immune cell populations in the spleen, and peripheral blood, quantifying the proportions of CD3^+^CD4^+^, CD3^+^CD8^+^, F4/80^+^CD11c^+^, and F4/80^+^CD206^+^ cells. (G) CD11c and CD206 levels under F4/80 gating after exogenous administration of FMT and BTX in *Card9*-knockout BMDMs. The data were compiled from at least three independent experiments and are presented as the means ± SEMs. **P* < 0.05, ***P* < 0.01, ****P* < 0.001 by Student’s unpaired two-tailed t test or one-way analysis of variance (ANOVA) with Tukey’s post hoc comparison.


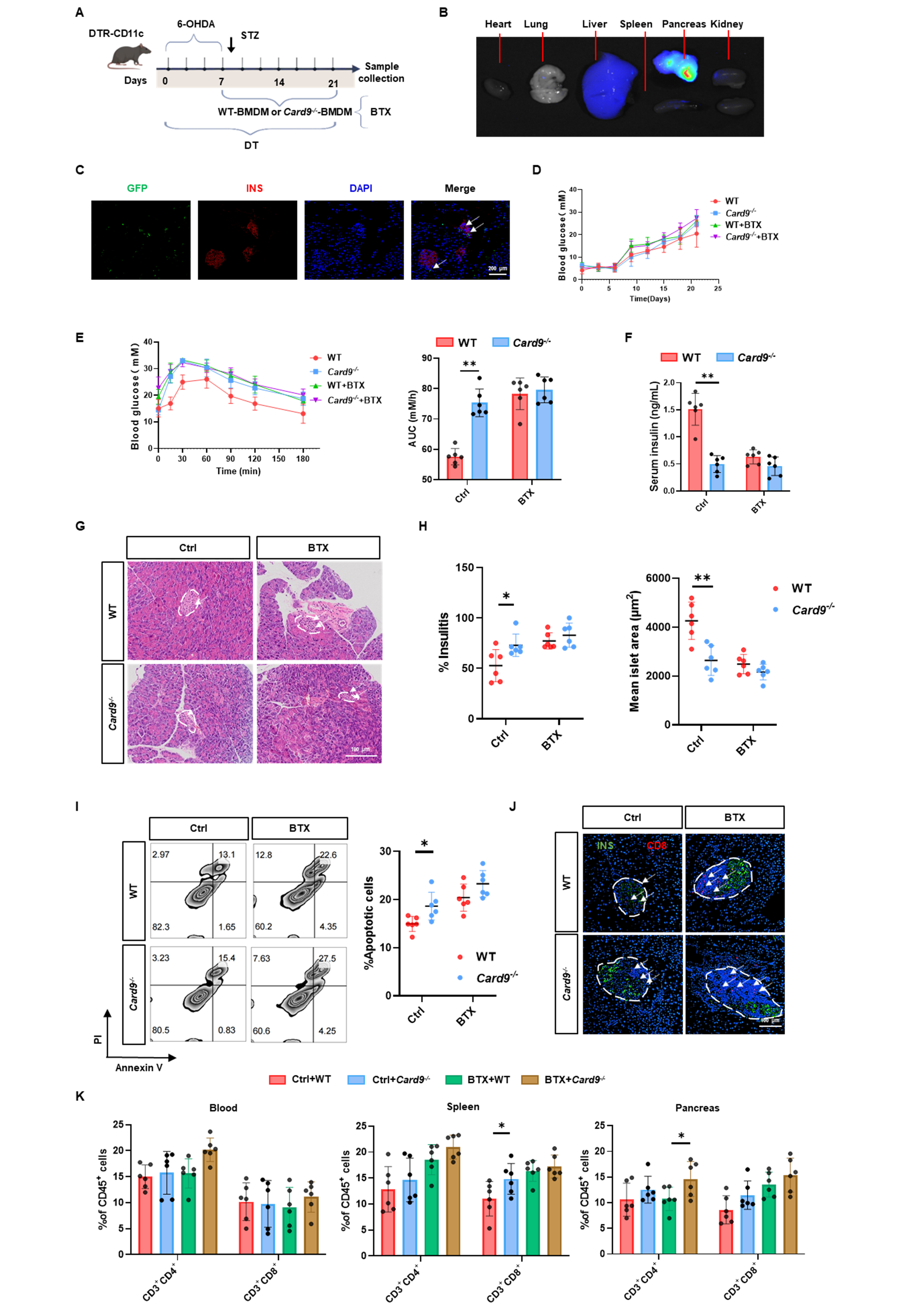


**Fig. S14 Effects of adoptive transfer of WT-BMDMs and Card9^-/-^ BMDMs on STZ-induced T1D mice with sympathetic denervation.** (A) Schematic diagram of the mouse experiment. DTR-CD11c mice were divided into four groups and injected with 6-OHDA to eliminate the sympathetic nervous system, followed by STZ-induced T1D modeling and adoptive transfer of WT-BMDMs or *Card9*^-/-^ BMDMs treated with PBS or BTX for observation. (B) Small animal imaging showing the in vivo distribution of BMDMs after adoptive transfer. (C) Immunofluorescence analysis of pancreatic tissues from STZ-induced mice following adoptive transfer of GFP-labeled BMDMs. (D) Blood glucose levels. (E) OGTT. (F) Plasma insulin levels. (G-H) Representative HE-stained sections of pancreatic tissue. (I) Apoptosis analysis of single pancreatic cells. (J) Representative immunofluorescence images of insulin and CD8 in pancreatic tissues. (K) Flow cytometry analysis of CD3^+^CD4^+^ and CD3^+^CD8^+^ cell populations in the spleen, whole blood, and pancreatic tissues of different groups of mice. The data were compiled from at least three independent experiments and are presented as the means ± SEMs. **P* < 0.05, ***P* < 0.01, ****P* < 0.001 by Student’s unpaired two-tailed t test or one-way analysis of variance (ANOVA) with Tukey’s post hoc comparison.


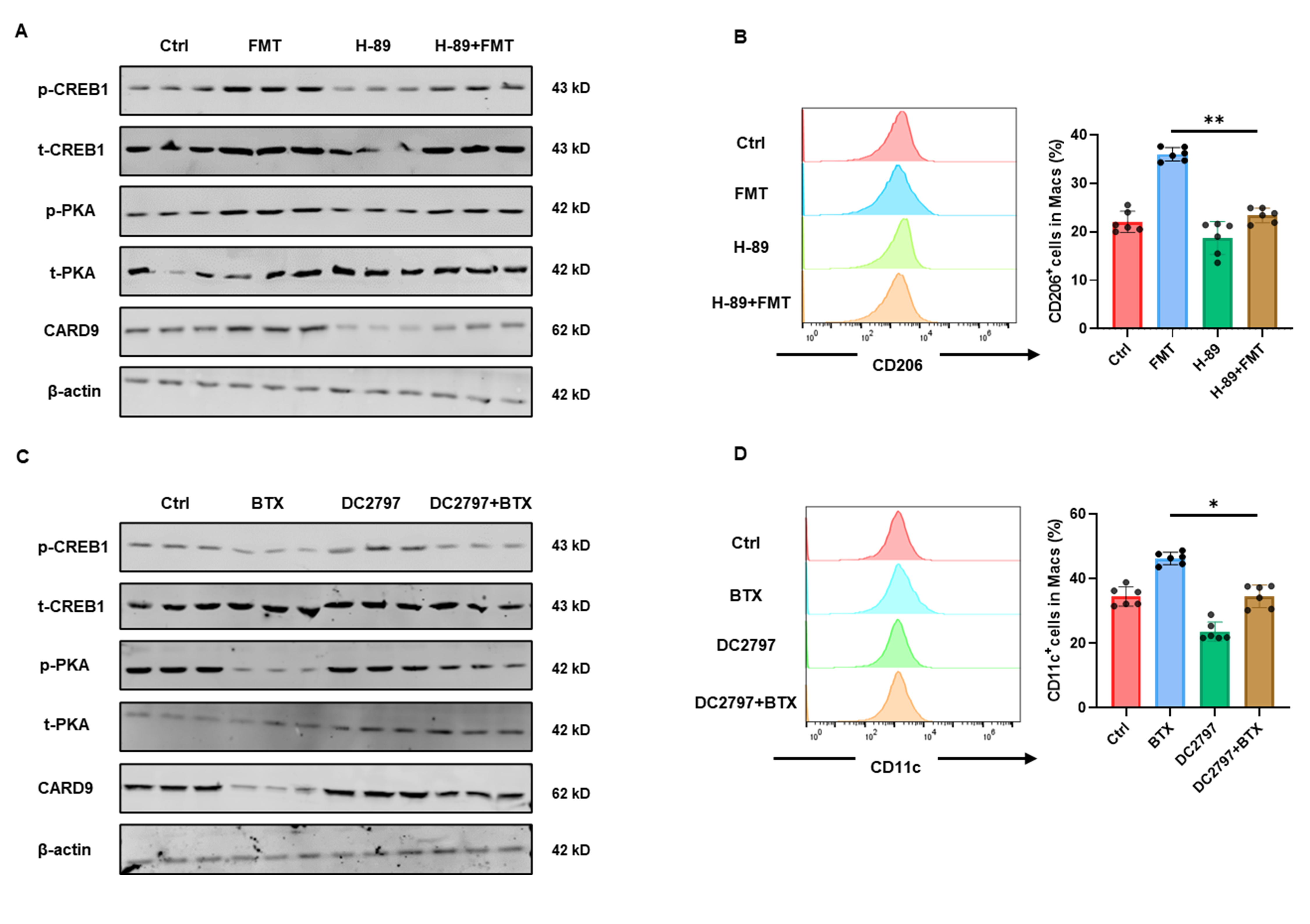


**Fig. S15 Effects of PKA/CREB1 signaling modulation on CARD9 expression and macrophage phenotypes.** (A) Expression levels of t-PKA, p-PKA, t-CREB1, p-CREB1, and CARD9 in BMDMs treated with FMT and H-89. (B) CD206 levels under the F4/80 gating in BMDMs after treatment with FMT and H-89. (C) Expression levels of t-PKA, p-PKA, t-CREB1, p-CREB1, and CARD9 in BMDMs treated with BTX and DC2797. (D) CD11c levels under the F4/80 gating in BMDMs treated with BTX and DC2797. The data were compiled from at least three independent experiments and are presented as the means ± SEMs. **P* < 0.05, ***P* < 0.01, ****P* < 0.001 by Student’s unpaired two-tailed t test or one-way analysis of variance (ANOVA) with Tukey’s post hoc comparison.


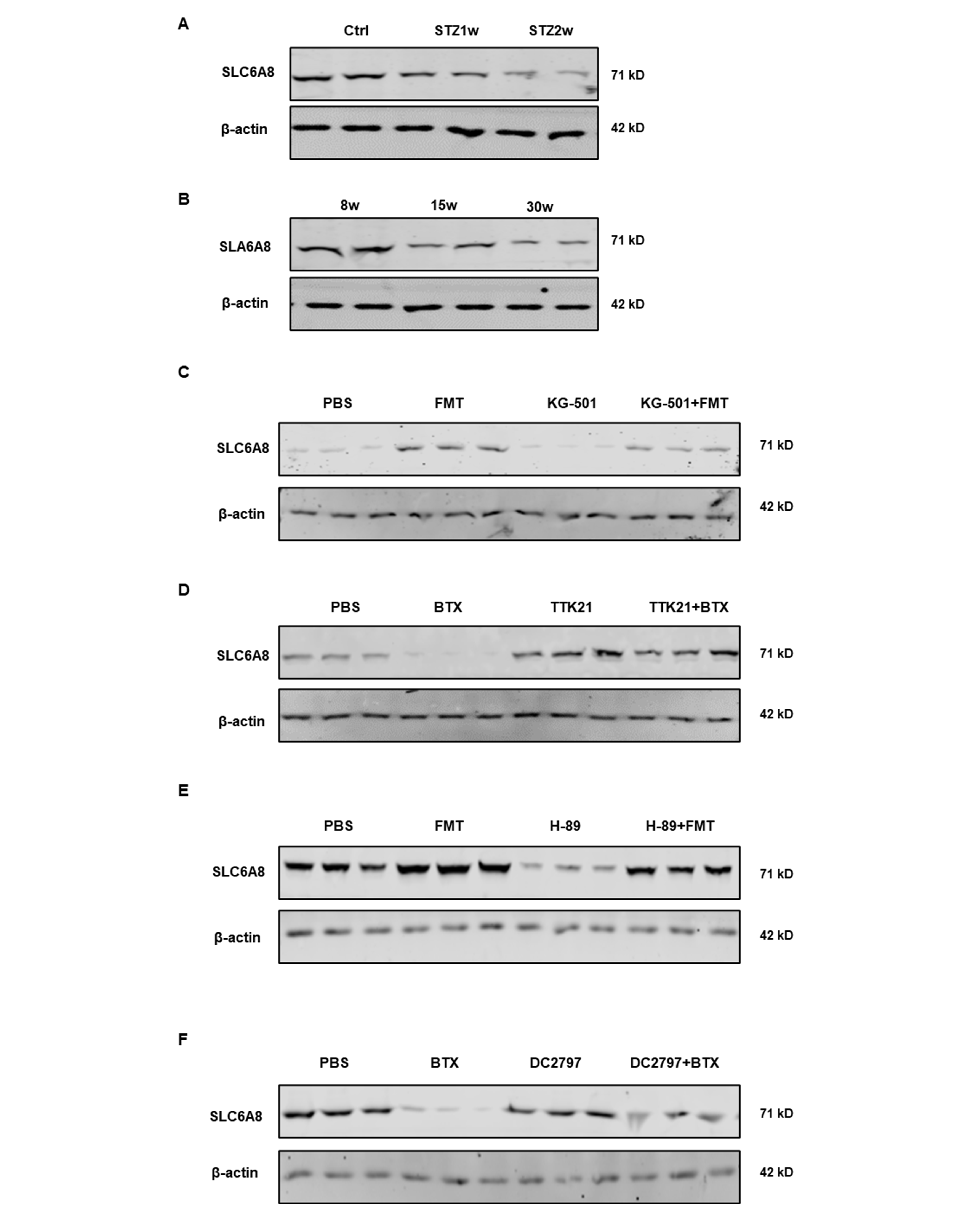


**Fig. S16 SLC6A8 expression in pancreatic tissues and BMDMs under different conditions.** (A) Western blot analysis of SLC6A8 expression in pancreatic tissues of STZ-induced T1D mice and normal mice. (B) Western blot analysis of SLC6A8 expression in pancreatic tissues of NOD mice at different ages. (D-G) Western blot analysis of SLC6A8 expression in BMDMs treated with FMT and KG-501 (D), BTX and TTK21 vector injections (E), FMT and H-89 (F), BTX and DC2797(G). The data were compiled from at least three independent experiments and are presented as the means ± SEMs. **P* < 0.05, ***P* < 0.01, ****P* < 0.001 by Student’s unpaired two-tailed t test or one-way analysis of variance (ANOVA) with Tukey’s post hoc comparison.

**Table S1 Patient information of the diabetes cohort.**

| **Sample ID** | **Gender** | **Age** | **Fasting blood glucose(mmol/L)** | **HbA1c(%)** |
| --- | --- | --- | --- | --- |
| A-1 | Female | 53 | 6.1 | 7.4 |
| A-2 | Male | 59 | 6.1 | 7.9 |
| A-3 | Male | 32 | 6.1 | 7.5 |
| A-4 | Male | 48 | 6.2 | 7 |
| A-5 | Male | 35 | 6.3 | 5.8 |
| A-6 | Male | 38 | 6.3 | 7.9 |
| A-7 | Female | 60 | 6.4 | 6.9 |
| A-8 | Male | 54 | 6.4 | 8.5 |
| A-9 | Male | 32 | 6.4 | 7.8 |
| A-10 | Male | 51 | 6.5 | 6.7 |
| A-11 | Female | 70 | 6.6 | 6.6 |
| A-12 | Female | 61 | 6.7 | 6.4 |
| A-13 | Male | 59 | 6.8 | 7 |
| A-14 | Female | 55 | 6.9 | 7.4 |
| A-15 | Male | 59 | 6.9 | 8.4 |
| A-16 | Female | 47 | 7 | 6.5 |
| A-17 | Male | 51 | 7 | 7.6 |
| A-18 | Male | 60 | 7.1 | 7.4 |
| A-19 | Male | 55 | 7.3 | 6 |
| A-20 | Male | 51 | 7.3 | 6.9 |
| A-21 | Female | 65 | 7.4 | 7.3 |
| A-22 | Male | 51 | 7.4 | 6.3 |
| A-23 | Male | 61 | 7.6 | 7.4 |
| A-24 | Male | 72 | 7.7 | 6 |
| A-25 | Male | 54 | 7.7 | 7.2 |
| A-26 | Male | 55 | 7.7 | 6.4 |
| A-27 | Female | 42 | 8 | 7.1 |
| A-28 | Male | 20 | 8.1 | 5.7 |
| A-29 | Male | 48 | 8.1 | 7.2 |
| A-30 | Male | 63 | 8.8 | 6.7 |
| A-31 | Male | 16 | 9.3 | 6.7 |
| A-32 | Male | 43 | 9.8 | 9.2 |
| A-33 | Male | 58 | 10 | 8.3 |
| A-34 | Male | 64 | 12.9 | 9.2 |
| A-35 | Male | 41 | 35.1 | 12.8 |
| A-36 | Male | 22 | 6.5 | 6.5 |
| A-37 | Female | 60 | 5.5 | 6.6 |
| A-38 | Male | 48 | 8.6 | 8.9 |
| A-39 | Female | 57 | 9.1 | 7.6 |

**Table S2 The details of the detection antibodies for western blot analysis and IF assay.**

| **Anti-body** | **Manufacturer** | **Catalogue numbers** |
| --- | --- | --- |
| Insulin | Cell Signaling Technology | 3014S |
| TH | Millipore | AB152 |
| CD86 | Cell Signaling Technology | 19589S |
| CD8 | Cell Signaling Technology | 98941S |
| GLU | Cell Signaling Technology | 2760S |
| Ki67 | Cell Signaling Technology | 9129S |
| F4/80 | Cell Signaling Technology | 30325S |
| CARD9 | Cell Signaling Technology | 12283S |
| ADRA1A | Proteintech | 19777-1-AP |
| ADRB2 | Proteintech | 29864-1-AP |
| t-SYK | Cell Signaling Technology | 13198S |
| p-SYK | Cell Signaling Technology | 2710S |
| t-PKA | Cell Signaling Technology | 4782S |
| p-PKA | Cell Signaling Technology | 4781S |
| t-CREB1 | Proteintech | 12208-1-AP |
| p-CREB1 | ABclonal | AP0903 |
| SLC6A8 | Proteintech | 20299-1-AP |
| Histon H3 | Cell Signaling Technology | 4499S |
| β-actin | Proteintech | 66009-1-Ig |
| Alexa Fluor® 488 Conjugate | Cell Signaling Technology | 4412S |
| Alexa Fluor® 594 Conjugate | Cell Signaling Technology | 8890S |

**Table S3 Sequences of Primer applied in Real-time Polymerase Chain Reaction**

| **Gene** |  | **Primer Sequences** |
| --- | --- | --- |
| *Gap43* | Forward | TGGTGTCAAGCCGGAAGATAA |
|  | Reverse | GCTGGTGCATCACCCTTCT |
| *Tubb3* | Forward | TAGACCCCAGCGGCAACTAT |
|  | Reverse | GTTCCAGGTTCCAAGTCCACC |
| *Stmn2* | Forward | GCCGCGCAACATCAACATC |
|  | Reverse | CCCCCTGGAGAAAGTTACCTT |
| *TH* | Forward | GTCTCAGAGCAGGATACCAAGC |
|  | Reverse | CTCTCCTCGAATACCACAGCC |
| *Card9* | Forward | TCCAGACGGAGAGCCGATTA |
|  | Reverse | CCTGGGTGAACTGTCTTCCAA |
| *Il-1β* | Forward | GCAACTGTTCCTGAACTCAACT |
|  | Reverse | ATCTTTTGGGGTCCGTCAACT |
| *Il-10* | Forward | GCTCTTACTGACTGGCATGAG |
|  | Reverse | CGCAGCTCTAGGAGCATGTG |
| *Tnf-α* | Forward | CCCTCACACTCAGATCATCTTCT |
|  | Reverse | GCTACGACGTGGGCTACAG |
| *Tgf-β* | Forward | CTCCCGTGGCTTCTAGTGC |
|  | Reverse | GCCTTAGTTTGGACAGGATCTG |
| *Adrb1* | Forward | CTCATCGTGGTGGGTAACGTG |
|  | Reverse | ACACACAGCACATCTACCGAA |
| *Adrb2* | Forward | GGGAACGACAGCGACTTCTT |
|  | Reverse | GCCAGGACGATAACCGACAT |
| *Adrb3* | Forward | GGCCCTCTCTAGTTCCCAG |
|  | Reverse | TAGCCATCAAACCTGTTGAGC |
| *Adra1a* | Forward | CTAAGGCCATTCTACTTGGGGT |
|  | Reverse | CGAGTGCAGATGCCGATGA |
| *ADRA1b* | Forward | CGAGTGCAGATGCCGATGA |
|  | Reverse | AACACAGGACATCAACCGCTG |
| *Adra1d* | Forward | AGTGGGTGTCTTCCTAGCC |
|  | Reverse | GCCTAGAACCTCCATAGTGGC |
| *Adra2a* | Forward | GTGACACTGACGCTGGTTTG |
|  | Reverse | CCAGTAACCCATAACCTCGTTG |
| *Adra2b* | Forward | TCTTCACCATTTTCGGCAATGC |
|  | Reverse | AGAGTAGCCACTAGGATGTCG |
| *Adra2c* | Forward | CTGTGGTGGGTTTCCTCATCG |
|  | Reverse | ACTTGCCCGAAGTACCAGTAG |
| *Bdnf* | Forward | TCATACTTCGGTTGCATGAAGG |
|  | Reverse | AGACCTCTCGAACCTGCCC |
| *Ngf* | Forward | CCAGTGAAATTAGGCTCCCTG |
|  | Reverse | CCTTGGCAAAACCTTTATTGGG |
| *Arg1* | Forward | CTCCAAGCCAAAGTCCTTAGAG |
|  | Reverse | AGGAGCTGTCATTAGGGACATC |
| *Cdnf* | Forward | CTTTTGCGCCGGGTTTTGTAT |
|  | Reverse | AGGGAGTTGTAGAATCGGTCTAA |
| *Slc6a8* | Forward | GCAGGGTGTGCATATCTCCAA |
|  | Reverse | TACCCCCACTCACATCAGTCA |
| *Gamt* | Forward | CACGCACCTGCAAATCCTG |
|  | Reverse | TACCGAAGCCCACTTCCAAGA |
| *Gatm* | Forward | GCTTCCTCCCGAAATTCCTGT |
|  | Reverse | CCTCTAAAGGGTCCCATTCGT |
| *Ckb* | Forward | AGTTCCCTGATCTGAGCAGC |
|  | Reverse | GAATGGCGTCGTCCAAAGTAA |
| *β-actin* | Forward | GGCTGTATTCCCCTCCATCG |
|  | Reverse | CCAGTTGGTAACAATGCCATGT |

**Table S4 Details of ELISA kits**

| **Analyte** | **Product Name** | **Source** | **Catalog Number** |
| --- | --- | --- | --- |
| Insulin (INS) | Human Insulin ELISA Kit | Abcam, UK | ab100578 |
| Insulin (INS) | Mouse Insulin ELISA Kit | Abcam, UK | ab277390 |
| Nerve Growth Factor (NGF) | Human NGF ELISA Kit | Abcam, UK | ab193760 |
| Brain-Derived Neurotrophic Factor (BDNF) | Human BDNF ELISA Kit | Jiangsu Meimian Industrial Co., Ltd | MM-0027H1 |
| Ciliary Neurotrophic Factor (CNTF) | Human CNTF ELISA Kit | Jiangsu Meimian Industrial Co., Ltd | MM-0016H1 |
| Leukemia Inhibitory Factor (LIF) | Human LIF ELISA Kit | Elabscience Biotechnology Co., Ltd | E-EL-H0094 |
| Arginase-1 (Arg-1) | Mouse Arginase-1 ELISA Kit | Abcam, UK | ab269541 |
| Norepinephrine (NA/NE) | NA/NE ELISA Kit | Elabscience Biotechnology Co., Ltd | E-EL-0047 |
| Dopamine (DA) | DA ELISA Kit | Elabscience Biotechnology Co., Ltd | E-EL-0046 |
| Epinephrine (EPI) | EPI ELISA Kit | Elabscience Biotechnology Co., Ltd | E-EL-0045 |
| Interleukin-1β (IL-1β) | Mouse IL-1β ELISA Kit | Elabscience Biotechnology Co., Ltd | E-EL-M0037 |
| Interleukin-10  (IL-10) | Mouse IL-10 ELISA Kit | Abcam, UK | ab255729 |
| TNF-α | Mouse TNF-α ELISA Kit | Abcam, UK | ab208348 |
| Cyclic Adenosine Monophosphate (cAMP) | cAMP ELISA Kit | Elabscience Biotechnology Co., Ltd | E-EL-0056 |
